# Differential associations of plasma biomarkers with Alzheimer's disease and small vessel disease: A multimodal imaging study

**DOI:** 10.1002/alz.71530

**Published:** 2026-06-07

**Authors:** Anna Dewenter, Katharina Bürger, Daniel Janowitz, Melina Paulus, Brigitte Nuscher, Fabian Hirsch, Benno Gesierich, Anna Steward, Lukas Frontzkowski, Sebastian N. Roemer‐Cassiano, Zeyu Zhu, Davina Biel, Madleen Klonowski, Gloria Biechele, Sophia Stoecklein, Michael Ewers, Marco Duering, Steffen Tiedt, Matthias Brendel, Nicolai Franzmeier, for the Alzheimer's Disease Neuroimaging Initiative (ADNI)

**Affiliations:** ^1^ Institute for Stroke and Dementia Research (ISD) University Hospital LMU Munich Munich Germany; ^2^ German Center for Neurodegenerative Diseases (DZNE) Munich Germany; ^3^ Division of Metabolic Biochemistry Biomedical Center (BMC) Faculty of Medicine Ludwig‐Maximilians‐Universität Munich Munich Germany; ^4^ Antaros Medical AB Mölndal Sweden; ^5^ Department of Nuclear Medicine University Hospital, LMU Munich Munich Germany; ^6^ Department of Neurology University Hospital, LMU Munich Munich Germany; ^7^ Department of Radiology University Hospital, LMU Munich Munich Germany; ^8^ Munich Cluster for Systems Neurology (SyNergy) Munich Germany; ^9^ Department of Psychiatry and Neurochemistry, The Sahlgrenska Academy, Institute of Neuroscience and Physiology University of Gothenburg Gothenburg Sweden

**Keywords:** Alzheimer's disease, biomarkers, cerebral small vessel disease, imaging, imaging biomarkers, mixed pathology, plasma biomarkers, precision medicine

## Abstract

**INTRODUCTION:**

We investigated how plasma biomarkers (phosphorylated tau 217 [ptau_217_], glial fibrillary acidic protein [GFAP], neurofilament light chain [NfL]) relate to imaging markers of small vessel disease (SVD) and Alzheimer's disease (AD), and cognition in memory clinic patients.

**METHODS:**

76 memory clinic patients underwent plasma biomarker assessment, neuropsychological testing, and 3T MRI. SVD burden was assessed using white matter hyperintensity (WMH) volume, mean skeletonized mean diffusivity (MSMD), and fiber density. AD‐related neurodegeneration was captured by AD‐signature cortical thickness and fiber‐bundle cross‐section. Findings were validated in 41 Alzheimer's Disease Neuroimaging Initiative (ADNI) participants with amyloid‐/tau‐positron emission tomography (PET).

**RESULTS:**

Associations varied between biomarkers. NfL showed strongest associations with SVD burden, ptau_217_ with AD‐related neurodegeneration, while GFAP was linked to both. SVD markers were associated with processing speed, whereas AD markers were most associated with memory.

**DISCUSSION:**

NfL relates to SVD burden, while ptau_217_ remains most sensitive to AD‐related biomarkers. GFAP's dual associations suggest overlapping biological processes. Together, coexisting SVD should be considered when interpreting plasma biomarkers in memory clinic patients.

## Highlights

- Phosphorylated tau 217 (ptau_217_) specifically reflects Alzheimer's disease (AD) pathology, even in the context of comorbid SVD
- Neurofilament light chain (NfL) and glial fibrillary acidic protein (GFAP) strongly relate to small vessel disease (SVD) burden and SVD‐typical cognitive deficits
- GFAP shows overlapping associations with both AD and SVD processes
- Diffusion MRI markers dissociate SVD‐ from AD‐related white matter injury and cognition
- Combining plasma and imaging biomarkers may reveal pathologies underlying cognitive deficits in memory clinics

## 1 BACKGROUND

Alzheimer's disease (AD) is the most common neurodegenerative disease in the elderly, characterized by amyloid‐beta (Aβ) plaques and neurofibrillary tau tangles, leading to neurodegeneration and ultimately dementia.^1^ Cerebral small vessel disease (SVD) is also common and affects the small perforating arterioles, capillaries, and venules, leading to brain parenchymal dysfunction. While the pathogenesis of SVD is incompletely understood, it is a major cause of vascular dementia and stroke.^2^ Post‐mortem studies demonstrated that pure AD or SVD rarely exist, with memory clinic patients typically exhibiting mixed AD and SVD pathology.^3^, ^4^ Unravelling the pathophysiology driving cognitive deficits is indispensable for patient‐centered diagnosis and treatment, underscoring the need for biomarkers that characterize the relative contributions of overlapping cerebrovascular and neurodegenerative processes.

Positron emission tomography (PET) and cerebrospinal fluid (CSF) are the gold standards for detecting Aβ and tau and for diagnosing AD in‐vivo.^5^ PET and CSF are invasive, and PET is expensive, entails radiation exposure, and has limited availability in clinical settings. Plasma AD biomarker panels are well‐suited for routine clinical use, with growing adoption due to better accessibility and cost‐effectiveness.^6^ In this regard, plasma phosphorylated tau 217 (ptau_217_) has shown high potential for diagnostic differentiation and prognostic assessment of AD.^7^, ^8^ Plasma neurofilament light chain (NfL) reflects neuroaxonal injury, while plasma glial fibrillary acidic protein (GFAP), an astrocytic cytoskeletal protein, indicates astrocyte activation and is often elevated during inflammatory processes. Both biomarkers capture broader neurobiological processes that intersect with AD pathophysiology.^9^, ^10^, ^11^, ^12^ Although NfL and GFAP have been studied as potential AD biomarkers, elevated levels are observed across multiple neurological conditions, including SVD, multiple sclerosis, and traumatic brain injury (TBI).^13^ GFAP is upregulated after central nervous system (CNS) trauma and is included in United States Food and Drug Administration (FDA) ‐approved blood tests for mild TBI.^14^ Further, GFAP rises acutely after small subcortical infarcts and normalizes over time, reflecting sensitivity to acute ischemic cerebrovascular injury.^15^ Similarly, NfL levels are elevated in individuals with TBI, multiple sclerosis, amyotrophic lateral sclerosis and SVD, reflecting unspecific neurodegeneration due to various pathophysiological processes.^13^ These observations raise important questions about the specificity of NfL and GFAP as AD biomarkers in memory clinic patients.

In parallel to plasma biomarkers, diffusion magnetic resonance imaging (MRI) detects white matter alterations in AD and SVD. Beyond conventional MRI metrics such as white matter hyperintensities (WMHs), advanced fixel‐based diffusion metrics quantify microstructural (fiber density [FD]) and macrostructural (fiber‐bundle cross‐section [FC]) white matter alterations. Our prior work showed that reduced FD indicates SVD, whereas reduced FC is characteristic of AD‐related neurodegeneration.^16^ Others linked frontal and thalamic fiber tract damage to SVD‐associated processing speed deficits,^17^, ^18^ while alterations in the parahippocampal cingulum and inferior longitudinal fasciculus seem to precede AD‐type fibrillar tau deposition and are linked to episodic memory impairment.^19^, ^20^ However, the capacity of advanced regional diffusion metrics to explain domain‐specific cognitive deficits in AD and SVD—and its comparison to plasma biomarkers—remains unclear.

To characterize biomarker patterns associated with cerebrovascular and neurodegenerative brain alterations, we investigated associations of plasma NfL, GFAP, and ptau_217_ with SVD‐ and AD‐biomarkers and examined the ability of plasma biomarkers to explain AD‐ vs. SVD‐typical cognitive deficits compared to advanced diffusion MRI markers. Specifically, we assessed (i) the contributions of AD‐ and SVD‐related brain alterations, as captured by imaging, to plasma biomarker levels, and (ii) the ability of imaging and plasma markers to explain domain‐specific cognitive impairment.

To this end, we included 76 memory clinic patients who underwent single‐molecule array (SIMOA) ‐based plasma biomarker quantification, comprehensive neuropsychological testing, and MRI. Imaging markers included AD‐signature cortical thickness, WMH volume, mean skeletonized mean diffusivity (MSMD), and advanced diffusion metrics (FD and FC) of strategic white matter tracts implicated in AD and SVD pathology. For independent validation, 41 participants from the Alzheimer's Disease Neuroimaging Initiative (ADNI) with additional gold‐standard amyloid‐ and tau‐PET imaging were included. Our primary goal was to investigate how plasma and imaging biomarkers relate to brain alterations typically observed in AD and SVD, and how these markers contribute to domain‐specific cognitive impairment.

**RESEARCH IN CONTEXT**

1. **Systematic review**: We reviewed the PubMed database to identify evidence on plasma biomarkers (phosphorylated tau 217 [ptau_217_], glial fibrillary acidic protein [GFAP], neurofilament light chain [NfL]) and diffusion magnetic resonance imaging (MRI) metrics in relation to Alzheimer's disease (AD) and cerebral small vessel disease (SVD). Prior studies show that ptau_217_ reflects AD pathology, while GFAP and NfL are elevated across multiple neurological conditions; however, the influence of coexisting SVD on these biomarkers in memory clinic patients remains insufficiently characterized.
2. **Interpretation**: We show that ptau_217_ remains AD‐specific despite comorbid SVD, whereas GFAP and NfL mainly reflect SVD‐related injury and cognitive deficits. GFAP's dual associations suggest shared inflammatory pathways across AD and SVD. Diffusion MRI further separates AD‐ from SVD‐related white matter changes. Together, these results refine plasma biomarker interpretation in mixed‐pathology settings.
3. **Future directions**: Future research should determine biomarker cutoffs accounting for SVD, assess longitudinal trajectories of plasma and diffusion markers in mixed pathology populations, and evaluate their relevance for clinical trial stratification.

## 2 METHODS

### 2.1 Participants

For exploration, we included data from the E‐Go Study, a cross‐sectional neuroimaging and biomarker investigation of memory clinic patients conducted at the Institute for Stroke and Dementia Research, LMU Hospital, Munich. Inclusion criteria were: age over 59 years, cognitive status of either cognitively normal (CN) or amnestic mild cognitive impairment (MCI), and fluency in German to enable neuropsychological testing. Participants underwent the comprehensive neuropsychological assessment using the Consortium to Establish a Registry for Alzheimer's Disease (CERAD)‐Plus battery, state‐of‐the‐art 3T MRI, and SIMOA‐based plasma biomarker assessment. In total, 96 individuals participated in the E‐Go Study. For the current investigation, we included only participants with complete MRI data, neuropsychological assessments, and plasma samples, resulting in 76 participants (44 cognitively normal, 32 with MCI). Plasma samples were collected on the day of neuropsychological testing for all participants. For most participants, neuropsychological testing coincided with the MRI visit (median 0 days, IQR 0–0 relative to MRI), whereas 12 participants underwent MRI 3–28 days after neuropsychological testing. In a subset of these participants, amyloid positivity was assessed through visual reads on amyloid‐PET or CSF ratios (Aβ_1‐42_/Aβ_1‐40_) by experts as part of routine clinical diagnostics.

For independent validation of our aims in a sample with additional amyloid‐ and tau‐PET, we incorporated data from participants of the ADNI. To ensure sufficient variability in cerebrovascular burden, analyses were restricted to participants of ADNI‐4, as earlier ADNI phases applied exclusion criteria that limited the inclusion of individuals with relevant cerebrovascular disease. Inclusion criteria for the current investigation were availability of plasma biomarker assessments, neuropsychological testing and acquisition of multi‐shell diffusion MRI, amyloid‐ and tau‐PET within 6 months before or after the plasma biomarker assessment. Due to this multimodal requirement, 41 participants met all inclusion criteria (25 cognitively normal, 14 with MCI, 2 with dementia).

### 2.2 Neuropsychological assessment

In E‐Go, participants underwent the comprehensive CERAD Plus test battery,^21^ conducted by trained neuropsychologists. The test battery included the following tests: Category Fluency, Boston Naming Test, Mini‐Mental State Examination score (MMSE), word list learning, letter fluency, Trail Making Test (TMT) matrix A and B. Individual test scores were converted to z‐scores and adjusted for age, sex, and education based on a healthy reference cohort (*n *= 1100). For SVD‐typical impairment, we a priori focused on performance in the TMT, as processing speed is often the earliest and sometimes the only affected cognitive domain in SVD.^22^ For AD‐typical impairment, we focused on the immediate recall score of the word list learning task that primarily reflects encoding and early retrieval processes.^23^

In ADNI, participants underwent comprehensive, standardized neuropsychological testing, which was summarized using pre‐established ADNI composite scores following ADNI protocols. For the present investigation, we included measures of memory (ADNI‐MEM),^24^ executive functioning without language involvement (ADNI‐EF2),^25^ language (ADNI‐LAN), and visuospatial functioning (ADNI‐VS),^26^ as well as global cognition using the 13‐item Alzheimer's Disease Assessment Scale‐Cognition (ADAS13),^27^ the Montreal Cognitive Assessment (MoCA), and the MMSE.

Briefly, ADNI‐MEM is a validated composite episodic memory score derived from item‐level data of the Rey Auditory Verbal Learning Test, Logical Memory, ADAS‐Cog word recall components, and MMSE recall items using confirmatory factor analysis. ADNI‐EF2 reflects executive functioning without language involvement and is based on the WAIS‐R Digit Symbol Substitution, Digit Span Backwards, Trail Making Test Parts A and B, Category Fluency, and Clock Drawing. ADNI‐LAN captures language abilities and includes the Boston Naming Test, verbal fluency measures, language‐related MMSE and ADAS‐Cog items, and MoCA language components. ADNI‐VS represents visuospatial functioning and is derived from clock copying, MMSE pentagon copying, and the ADAS‐Cog constructional praxis item. Following a similar approach as in E‐Go, we a priori focused on ADNI‐EF2 as a proxy for (nonverbal) executive functioning, reflecting the cognitive profile typically affected in SVD, and on ADNI‐MEM as a proxy for episodic memory performance, often impaired in AD.

### 2.3 MRI

### 2.4 MRI acquisition and MRI markers

In the E‐Go study, MRI data were acquired on a single 3T scanner (Magnetom Prisma with 64‐channel head/neck coil; SIEMENS Healthineers, Erlangen, Germany). Relevant sequences included 3D T1‐weighted (Magnetization Prepared RApid Gradient Echo [MPRAGE]), 3D fluid‐attenuated inversion recovery (FLAIR), and a multi‐shell diffusion MRI sequence. The diffusion protocol used a multi‐band echo planar imaging sequence with a repetition time of 3800 ms, echo time of 105 ms, 30 diffusion directions at *b* = 1000 s/mm^2^, 60 directions at *b* = 2000 s/mm^2^, and 10 *b* = 0 images (multi‐band factor = 3). Additionally, one *b* = 0 image with reversed phase‐encoding direction was acquired to correct for susceptibility‐induced distortions. In the ADNI study, MRI scans were also performed on 3T Siemens scanners (SIEMENS Healthineers, Erlangen, Germany) using comparable sequences (3D T1w MPRAGE, 3D FLAIR, and multi‐shell diffusion MRI). The diffusion sequence was scanned with a repetition time of 3800 ms, echo time of 105 ms, 29 diffusion directions at *b* = 1000 s/mm^2^, 38 at *b* = 2000 s/mm^2^, and 13 *b* = 0 images.

Imaging markers were in both samples assessed in the same manner. WMH volume was assessed with a deep learning‐based segmentation algorithm (https://github.com/miac‐research/MARS‐WMH) as already used previously.^16^, ^28^

Cortical thickness for Desikan–Killiany atlas regions of interest (ROIs) and total intracranial volume were assessed based on T1‐weighted MRI scans using the CAT12 toolbox (https://neuro‐jena.github.io/cat/).^29^ To obtain a global measure of AD‐related neurodegeneration, we assessed and averaged cortical thickness of individual ROIs of the Desikan–Killiany Atlas that are part of a pre‐established AD signature ROI.^30^, ^31^

### 2.5 Diffusion MRI processing

Preprocessing steps of diffusion MRI included visual quality control, Marchenko–Pastur principal component analysis‐based denoising, Gibbs artefact removal, and dynamic correction for susceptibility‐induced distortions, eddy current‐induced distortions, as well as head motion using tools from MRtrix3 (www.mrtrix.org/, version 3.0.2, dwidenoise,^32^, ^33^, ^34^, ^35^ mrdegibbs^35^, ^36^) and the Functional Magnetic Resonance Imaging of the Brain Software Library (FSL, version 6.0.1, topup,^37^, ^38^ eddy^39^ including state‐of‐the art replacement of outliers,^40^ usage of the slice‐to‐volume motion model^41^ and susceptibility‐by‐movement correction^42^). Due to unavailability of an unweighted diffusion image with reversed phase‐encoding in the ADNI sample, we used Synb0‐DISCO^43^ to synthesize an unweighted diffusion image without susceptibility‐induced distortion from the T1‐weighted image. Other than this single step, preprocessing was kept identical.

Mean skeletonized mean diffusivity (MSMD) was assessed as outlined previously.^44^, ^45^

**FIGURE 1:**
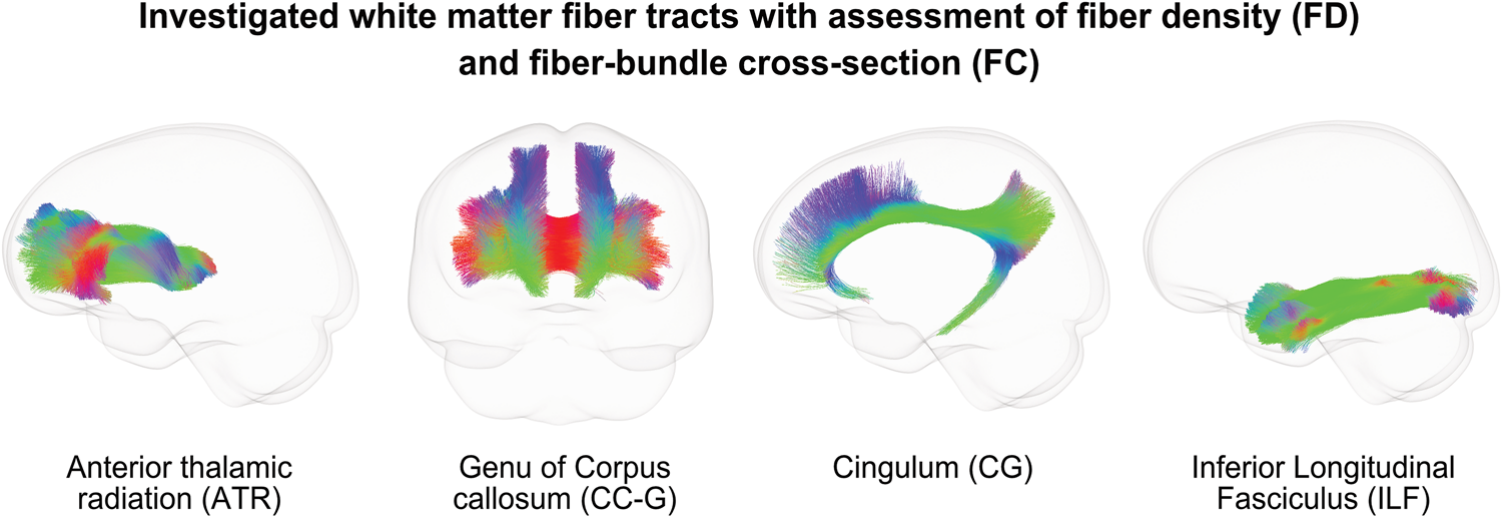
Strategic white matter fiber tracts. Fiber density and fiber‐bundle cross‐section were assessed in four white matter tracts: ATR, CC‐G, CG, and ILF. White matter tracts were segmented with TractSeg, a deep learning‐based segmentation tool. ATR, anterior thalamic radiation; CC‐G, genu of the corpus callosum; CG, cingulum; ILF, inferior longitudinal fasciculus.

For fixel‐based analysis, we followed the developers’ recommended pipeline and applied multi‐shell multi‐tissue constrained spherical deconvolution to compute fiber orientation distributions (FODs).^46^, ^47^ Each sample was processed independently. Diffusion MRI data were corrected for bias fields. Response functions were estimated for each participant using the ‘dhollander’ algorithm,^48^ based on which the mean response functions were computed. Remaining steps included upsampling to 1.25 voxel size, estimation of the fiber‐orientation distributions using the group response functions (“msmt_csd” algorithm) and intensity normalization. Next, a study‐specific FOD template was calculated using data from 30 representative individuals (sex‐ and gender‐balanced). Subject‐specific FOD images were registered to the FOD template and segmented to estimate fixels and their apparent FD and FC. Next, we used TractSeg to segment the FOD template into anatomically well‐established white matter tracts.^49^ To minimize the number of comparisons and since we had no primary hypothesis given laterality, we averaged tract measures for left and right hemispheres. Given their strategic significance for SVD‐ and AD‐typical cognitive impairment,^50^, ^51^, ^52^ we a priori focused on four white matter tracts (Figure 1): anterior thalamic radiation (ATR) and the genu of the corpus callosum (CC‐G), strategic regions of SVD‐related cognitive deficits^18^, ^51^; the cingulum (CG) and the inferior longitudinal fasciculus (ILF), key regions affected early in the AD cascade. We then assessed the average in FD and FC of the respective fiber tract in each study participant. For clarity, we use the term “fixel‐fiber tract combinations” to describe these tract‐wise aggregates of fixel‐based measures throughout the manuscript. Additional exploratory fiber tract analyses are reported in the supplementary, following our previous approach.^16^

### 2.6 PET

### 2.7 PET acquisition and analysis

PET data were only available in ADNI. PET imaging followed intravenous injection of ^18^F‐radiolabeled tracers, including Florbetapir or Florbetaben for amyloid‐PET and Flortaucipir for tau‐PET, using acquisition windows consistent with ADNI protocols. MRI and PET data were processed using an established in‐house pipeline. In short, T1‐weighted MRIs were bias‐corrected, segmented, and normalized to Montreal Neurological Institute (MNI) space using CAT12. PET images were realigned, averaged, and registered to MRI scans; standardized uptake value ratios (SUVRs) were computed using cerebellar reference regions and harmonized to centiloid units for amyloid‐PET. Tau‐PET SUVR maps were normalized to MNI space, and regional values were extracted in an established temporal meta ROI.^53^

### 2.8 Plasma biomarkers and apolipoprotein E genotyping

In E‐Go, ptau_217_, GFAP, and NfL were quantitatively determined using commercial SIMOA kits (ALZpath Simoa pTau_217_ Early Access Kit; Neurology 4‐Plex E, Quanterix) according to the manufacturer's instructions. All samples were analyzed blinded to clinical information and measured on the Simoa HD‐X analyzer (Quanterix). Genotyping was performed with the Illumina Global Screening Array (GSA‐MDv4). Apolipoprotein E (ApoE) status was subsequently determined based on the genotypes of the two key single nucleotide polymorphisms (SNPs), rs429358 and rs7412, extracted from the array data.

In ADNI, ptau_217_ was measured using the Fujirebio Lumipulse G1200 platform (Lumipulse G pTau217 Plasma), while GFAP and NfL were assessed with the Quanterix Neurology 4‐Plex E Advantage V1 assay on a SIMOA HD‐X platform. Details of the plasma biomarker procedures have been published elsewhere.^54^ The ApoE status for each individual was determined using DNA from blood samples.

### 2.9 Statistical analyses

All statistical analyses were performed in R (version 3.6.1, R core team).^55^ The statistical significance level was set at *α* < 0.05.

To compare between groups defined by cognitive status (CN, MCI, dementia) with respect to demographic characteristics, vascular risk factors, and MRI markers, we used chi‐squared (*χ* ^2^) tests (for categorical variables) and non‐parametric Wilcoxon rank sum tests and Kruskal–Wallis tests (for numeric variables), as appropriate. Since fixel metrics have shown to be significantly influenced by head size,^56^ we first regressed out the effect of intracranial volume as recommended by the developers and conducted subsequent analysis on residual fixel metrics corrected for head size. For this, we computed simple linear regression models with the log‐transformed intracranial volume as independent and the respective fixel‐fiber tract combination as dependent variable (‘lm’, ‘stats’ package).

To assess the primary determinants of plasma biomarkers with respect to aim (i), we performed simple linear regression and random forest regression analyses. To reduce the number of predictors for the random forest regression, we first performed principal component analysis on the FD and FC of the four investigated tracts (“prcomp”, “stats” package). For simple linear regression analyses, we explored associations between plasma biomarkers (dependent variable) and fixel‐fiber tract combinations, the first principal component of FD, the first principal component of FC, MSMD, WMHvolume and cortical thickness in an AD signature ROI (independent variable; “lm”, “stats” package). In ADNI, we additionally incorporated measures of global centiloids and temporal meta tau‐PET SUVR. Effect sizes were captured with the adjusted *R* ^2^. Since we focused primarily on effect sizes rather than statistical significance, we did not apply multiple comparison correction. Given that the investigated SVD and AD markers are highly intercorrelated, we further employed random forest regression analyses to assess the relative importance of the imaging markers for explaining the levels of each plasma biomarker. By simultaneously including markers commonly associated with cerebrovascular and neurodegenerative processes in multivariable models, this approach allowed estimation of their relative contributions within a mixed‐pathology framework. Specifically, we used multivariable random forest regression with conditional inference trees to infer the variable importance (R package “party”, version 1.3.3; number of trees = 1501, mtry = 3). We repeated random forest regression for each model (with cross‐validation) 100 times to determine a point estimate of the mean variable importance and a 95% confidence interval.

For aim (ii), we systematically assessed associations between biomarkers and performance in the neuropsychological test battery using simple linear regression analyses (neuropsychological performance as dependent variable, biomarker as independent variable). Here, we also focused on effect sizes (adjusted *R* ^2^) when interpreting results. In E‐Go, we a priori focused on performance in the TMT‐B and word list learning paradigm as SVD‐ and AD‐typical cognitive deficit, respectively. In ADNI, we focused on the ADNI‐EF2 composite score for SVD‐typical deficits, and on the ADNI‐MEM composite score for AD‐typical deficits. However, we also report on associations with other tests in the supplementary materials to exploratorily examine cognitive domains that are not typically linked to the respective diseases. In addition, we performed random forest regression analyses to assess the relative importance of imaging and plasma biomarkers for explaining neuropsychological performance using the same hyperparameters as above. Primary linear regression analyses were conducted as single‐predictor models (one imaging or plasma biomarker at a time) without additional covariate adjustment, as the objective was to compare the relative explanatory performance of individual markers using adjusted *R* ^2^. Cognitive test scores were standardized to age‐, sex‐, and education‐adjusted z‐scores prior to analysis; therefore, no additional demographic covariates were included in models predicting cognitive performance. Analyses were performed separately for each sample.

### 2.10 Data availability

Anonymized data of the E‐Go study will be made available upon request to the corresponding author and after permission of the regulatory bodies. ADNI data are publicly available at https://ida.loni.usc.edu/ upon registration.

## 3 RESULTS

Sample demographics and summary statistics are displayed in Table 1. Race and ethnicity data were available only for ADNI participants, among whom 90% identified as White, 5% as Asian, and 5% as Black or African American; 7% identified as Hispanic or Latino.

**TABLE 1:** Demographics and summary statistics of the sample.

	E‐Go			ADNI			
Parameter	CN (n = 44)	MCI (n = 32)	*p*‐Value	CN (n = 25)	MCI (n = 14)	DEM (n = 2)	*p*‐Value
Demographic characteristics							
Age [years], median (IQR)	74 (10.25)	75 (13.25)	0.297	71.3 (8.10)	72.2 (11.7)	80.2 (2.65)	0.230
Female, *n* (%)	18 (41)	10 (31)	0.417	15 (60)	5 (36)	1 (50)	0.346
ApoE‐ε4 carrier, *n* (%)^a^	11/30 (37)	8/21 (38)	1.0	8 (32)	6 (43)	2 (100)	0.155
Education [years], median (IQR)	14 (5)	13 (5.25)	0.607	18 (2.0)	16 (5.5)	16 (4.0)	0.147
Vascular risk factors, *n* (%)							
Hypertension	25 (57)	17 (53)	1.000	7 (28)	7 (50)	2 (100)	0.078
Diabetes mellitus	2 (5)	7 (22)	0.045	2 (8)	2 (14)	0 (0)	0.730
Hypercholesterolemia	26 (59)	17 (53)	0.685	5 (20)	7 (50)	2 (100)	0.022
Current or past smoking	17 (39)	15 (47)	0.546	8 (32)	4 (29)	0 (0)	0.631
Atrial fibrillation	2 (5)	5 (16)	0.195	0 (0)	1 (7)	0 (0)	0.372
MRI, median (IQR)							
WMH volume^b^, %	0.923 (1.776)	2.137 (8.130)	0.053	1.267 (1.942)	1.88 (6.676)	11.72 (4.479)	0.062
SVD score	1 (1)	1 (2)	0.424	1 (1)	2 (2)	2 (1)	0.233
AD cortical thickness	2.631 (0.164)	2.495 (0.150)	0.001	2.683 (0.085)	2.623 (0.223)	2.495 (0.185)	0.190
PET, median (IQR)							
amyloid‐PET	–	–	–	9.7 (15.94)	33.46 (80.07)	107.53 (16.27)	0.032
tau‐PET	–	–	–	1.154 (0.112)	1.138 (0.234)	1.606 (0.318)	0.171
Plasma biomarkers, median (IQR)							
ptau_217_ (pg/mL)	0.232 (0.197)	0.549 (0.492)	< 0.001	0.082 (0.043)	0.158 (0.445)	0.572 (0.206)	0.013
NfL (pg/mL)	17.350 (8.275)	21.950 (16.924)	0.003	14.70 (7.90)	15.60 (16.65)	34.35 (0.95)	0.059
GFAP (pg/mL)	105.500 (76.475)	136 (75.75)	0.008	110.10 (57.02)	130.05 (92.05)	303.65 (26.85)	0.033
Clinical scores							
MMSE	30 (1.0)	27 (2.25)	< 0.001	30 (1.0)	28 (2.75)	25 (1.00)	0.003

^a^Please note that ApoE‐ε4 carrier status was only available in 30/44 CN and 21/32 MCI individuals in E‐Go.

^b^Normalized to the intracranial volume.

Abbreviations: AD, Alzheimer's disease; ADNI, Alzheimer's Disease Neuroimaging Initiative; ApoE, apolipoprotein E; CN, cognitively normal; DEM, dementia; GFAP, glial fibrillary acidic protein; IQR, interquartile range; MCI, mild cognitive impairment; MMSE, Mini‐Mental State Examination; MRI, magnetic resonance imaging; PET, positron emission tomography; SVD, small vessel disease; WMH, white matter hyperintensity.

In the E‐Go sample, individuals with MCI showed a higher prevalence of diabetes, reduced cortical thickness in the AD signature ROI, increased levels of ptau_217_, NfL, and GFAP, and lower MMSE scores compared to the CN group (*p *< 0.05). Across both diagnostic groups, 37% of our participants with available DNA had at least one ApoE‐ε4 allele. Amyloid status was assessed in 45 of the 76 participants, with 17 (38%) classified as amyloid positive. Among those assessed, 7 of 20 were positive on routine CSF assessment (2 CN, 5 MCI) and 10 of 15 were positive on amyloid‐PET visual read (1 CN, 9 MCI).

In the ADNI sample, centiloid values differed significantly across diagnostic groups, with the highest values observed in participants with dementia (*p *= 0.032). The incidence of hypercholesterolemia also rose across diagnostic groups (*p *< 0.05). Plasma levels of ptau_217_ and GFAP increased, while MMSE scores declined across the diagnostic spectrum from CN to MCI to dementia (all *p *< 0.05). NfL levels showed a similar upward trend, though not reaching statistical significance (*p *= 0.059).

### 3.1 NfL and GFAP levels are highly associated with SVD burden, while ptau_217_ is associated with markers of AD

To address aim (i), we assessed the main determinants of plasma biomarker levels using simple linear regression and random forest regression analyses.

In the E‐Go sample, NfL showed the strongest associations with SVD‐related imaging markers, including FD of the CC‐G and mean skeletonized mean diffusivity (MSMD; both *R* ^2^_adj_. = 0.21). These were followed by the first principal component of FD (*R* ^2^_adj_. = 0.20), anterior thalamic radiation (ATR; *R* ^2^_adj_. = 0.19), global WMH volume (*R* ^2^_adj_. = 0.14), and FD of the CG and ILF with R^2^_adj_. of 0.13 and 0.10, respectively. Associations with AD‐signature cortical thickness were weaker (*R* ^2^_adj_. = 0.06). GFAP was primarily related to SVD markers, particularly FD PC1 (*R* ^2^_adj_. = 0.20), MSMD, and FD of CC‐G and CG (all *R* ^2^_adj_.≈0.18), but also showed a notable association with AD‐signature cortical thickness (*R* ^2^_adj_. = 0.17). In contrast, ptau_217_ was most strongly associated with AD‐signature cortical thickness (*R* ^2^_adj_. = 0.21), whereas associations with SVD metrics including FD PC1, MSMD, and CG FD were markedly weaker (all *R* ^2^_adj_.≈0.05), indicating higher specificity of ptau_217_ for AD‐related neurodegeneration.

**FIGURE 2:**
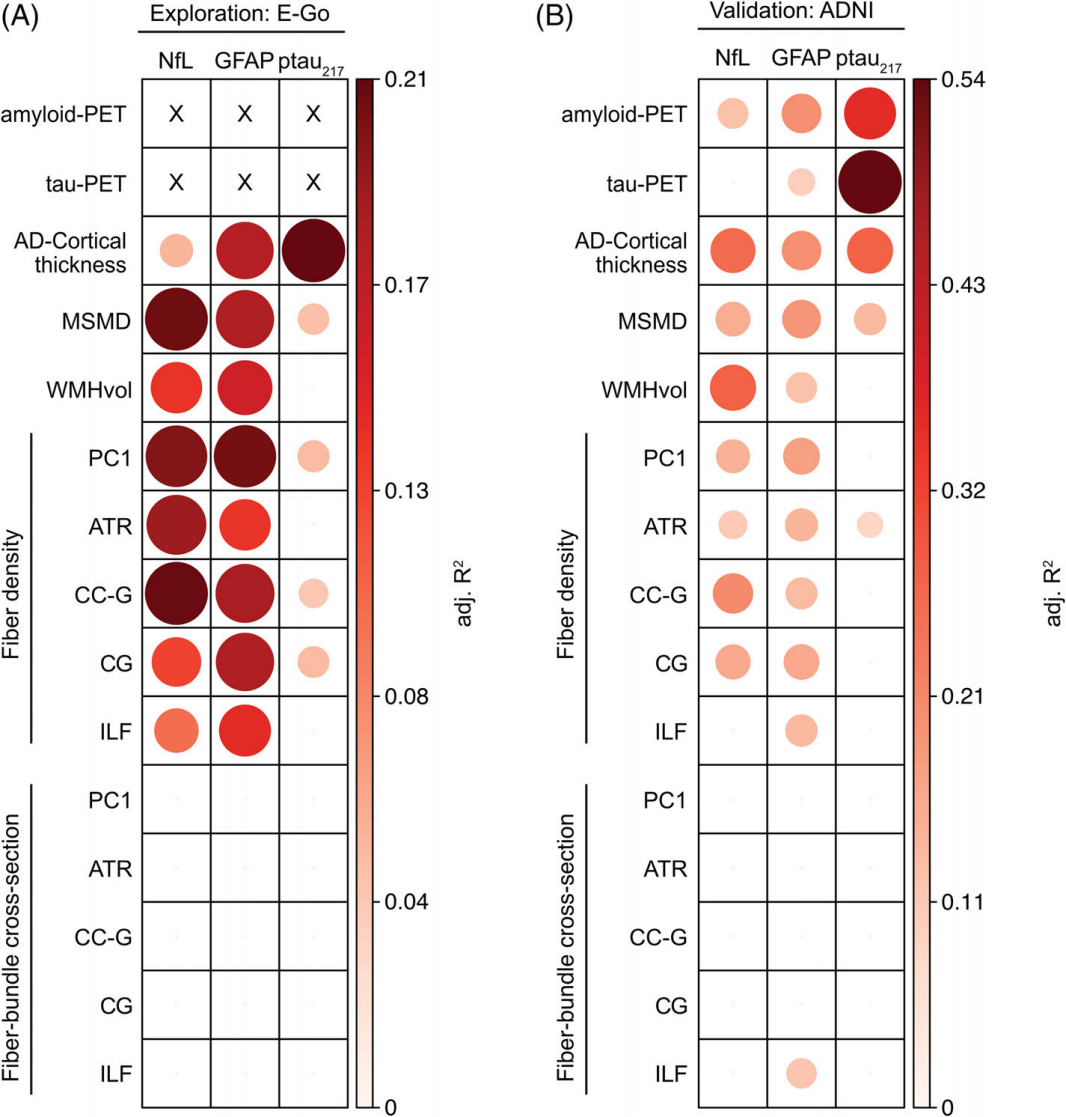
Associations between plasma and imaging markers. Results from simple linear regression analyses obtained in (A) E‐Go and for independent validation in (B) ADNI. Effect sizes (adjusted *R* ^2^) are represented by color (*p *< 0.05). ADNI, Alzheimer's Disease Neuroimaging Initiative; ATR, anterior thalamic radiation; CC‐G, genu of the corpus callosum; CG, cingulum; GFAP, glial fibrillary acidic protein; ICV, intracranial volume; ILF, inferior longitudinal fasciculus; MSMD, mean skeletonized mean diffusivity; NfL, neurofilament light chain; PC1, first principal component; ptau_217_, phosphorylated tau 217; WMHvol, white matter hyperintensity volume (normalized to ICV).

In ADNI, similar patterns emerged. NfL was predominantly linked to SVD‐related white matter damage, most strongly to global WMH volume (*R* ^2^_adj_. = 0.28), followed by AD‐signature cortical thickness (*R* ^2^_adj_. = 0.26) and SVD‐related diffusion metrics (CC‐G FD, *R* ^2^_adj_. = 0.21; CG *R* ^2^_adj_. = 0.16; MSMD *R* ^2^_adj_. = 0.16; FD‐PC1 *R* ^2^_adj_. = 0.15), with weaker associations with amyloid‐PET (*R* ^2^_adj_. = 0.12). GFAP showed strong associations with both amyloid‐PET and AD‐signature cortical thickness (both *R* ^2^_adj_. = 0.20), as well as with SVD markers including MSMD (*R* ^2^_adj_. = 0.19) and FD metrics across several tracts (FD‐PC1 *R* ^2^_adj_. = 0.17; CG *R* ^2^_adj_. = 0.16; ATR *R* ^2^_adj_. = 0.14; ILF and CC‐G, *R* ^2^_adj_. = 0.13). Ptau_217_ was most strongly linked to AD‐specific pathology, including temporal meta tau‐PET SUVR (*R* ^2^_adj_. = 0.54) and global amyloid‐PET (*R* ^2^_adj_. = 0.36), followed by a moderate association with AD‐signature cortical thickness (*R* ^2^_adj_. = 0.28), whereas relationships with diffusion‐ or SVD‐related markers were weak (*R* ^2^_adj_.≤0.13). A full list of assessed variables can be found in Figure 2 and Table e‐S1.

**FIGURE 3:**
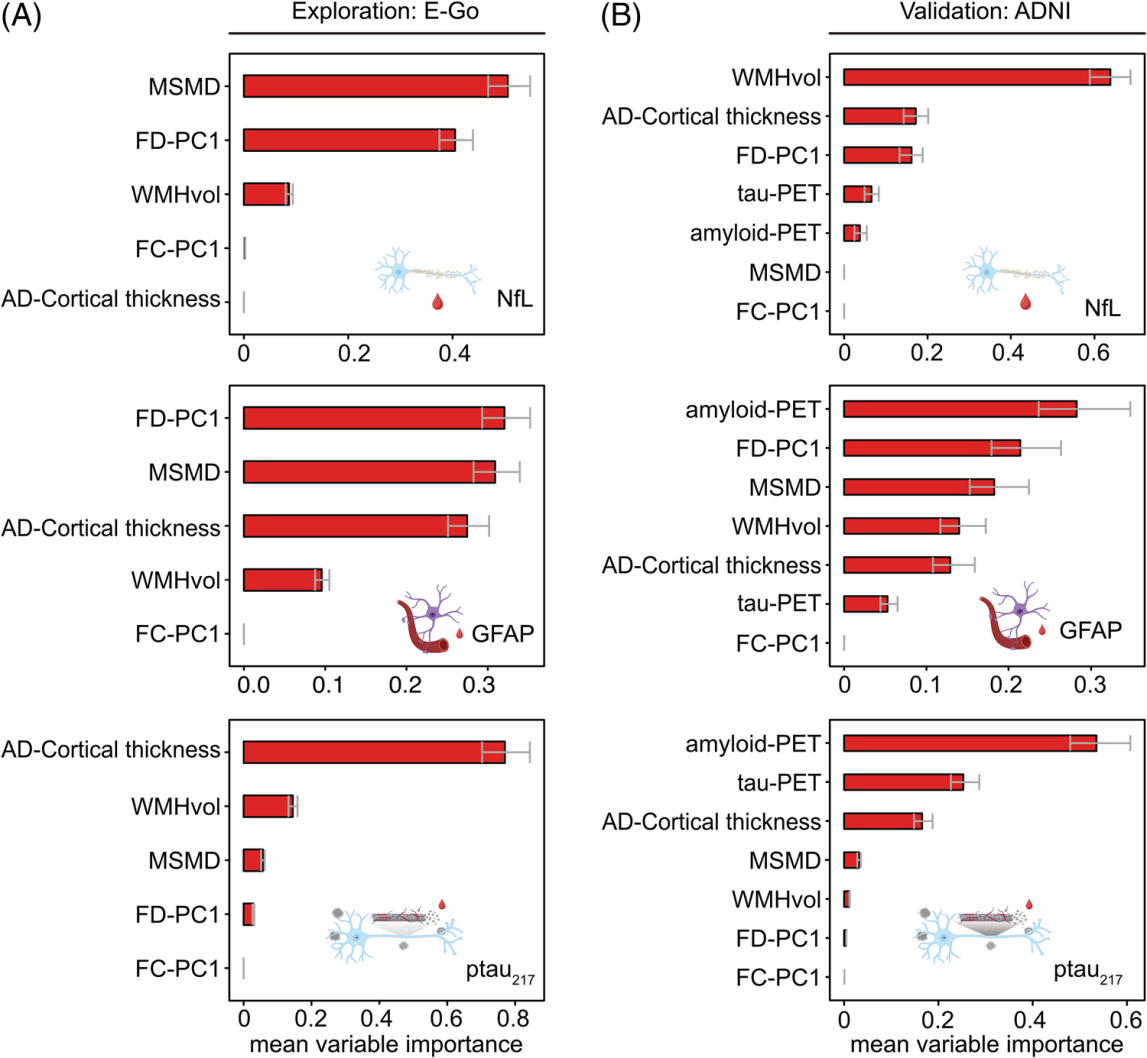
Random forest regression analyses showing the relative variable importance of imaging markers for the respective plasma biomarker in (A) E‐Go and (B) ADNI. Plots display point estimates with 95% confidence intervals for the conditional variable importance. ADNI, Alzheimer's Disease Neuroimaging Initiative; FC‐PC1, first principal component of fiber‐bundle cross‐section; FD‐PC1, first principal component of fiber density; GFAP, glial fibrillary acidic protein; ICV, intracranial volume; MSMD, mean skeletonized mean diffusivity; NfL, neurofilament light chain; ptau_217_, phosphorylated tau 217; WMHvol, white matter hyperintensity volume (normalized to ICV).

To examine the relative contributions of imaging and plasma biomarkers in a multivariable context, we conducted random forest regression analyses (Figure 3). In E‐Go, for NfL, MSMD had the highest relative variable importance (50.58%), followed by FD PC1 (40.49%). WMH volume and FC contributed minimally (8.65% and 2.90%, respectively), while AD‐signature cortical thickness did not contribute meaningfully (0%). GFAP showed a similar pattern, with the first principal component of FD contributing 32.06%, MSMD 30.09%, and AD‐signature cortical thickness 27.46%, whereas WMH volume and FC contributed 10.51% and 0%, respectively. For ptau_217_, AD‐signature cortical thickness dominated the contribution (76.97%), followed by WMH volume (14.47%), while FD PC1, MSMD, and FC contributed minimally (0%–5.68%).

In ADNI, NfL was primarily influenced by WMH volume (59.37%), with moderate contributions from AD‐signature cortical thickness (15.98%) and FD PC1 (15.03%), while tau‐PET (6.10%) and amyloid‐PET (3.50%) contributed minimally. GFAP exhibited a mixed profile, with amyloid‐PET contributing 28.24%, FD PC1 21.40%, MSMD 18.23%, WMH volume 13.97%, and AD‐signature cortical thickness 12.89%, whereas tau‐PET and FC‐PC1 contributed negligibly (< 0.06%). For ptau_217_, amyloid‐PET dominated (53.52%), followed by temporal meta tau‐PET (25.27%) and AD‐signature cortical thickness (16.54%), with SVD markers contributing minimally (< 5%).

In summary, across both cohorts, NfL increases were most associated with SVD‐related imaging markers, GFAP with a mixed profile of SVD‐ and AD‐related brain alterations, and ptau_217_ predominantly with AD‐specific imaging markers, confirming its high sensitivity and specificity for AD pathology.

### 3.2 NfL, GFAP, and FD are highly associated with SVD‐typical cognitive impairment, while ptau_217_ and FC are associated with AD‐typical cognitive impairment

In the E‐Go cohort, for the TMT‐B, assessing processing speed typically impaired in SVD, the strongest associations were observed with NfL (*R* ^2^_adj_. = 0.13), followed by MSMD (*R* ^2^_adj_. = 0.12), CC‐G FD (*R* ^2^_adj_. = 0.11), FD PC1 (*R* ^2^_adj_. = 0.10), CG FD (*R* ^2^_adj_. = 0.09), GFAP (*R* ^2^_adj_. = 0.07), and ATR FD (*R* ^2^_adj_. = 0.06). Notably, ptau_217_ and AD‐signature cortical thickness were not significantly associated with processing speed performance.

For word list learning, representing a domain often impaired in AD, the strongest associations were observed with AD‐signature cortical thickness (*R* ^2^_adj_. = 0.19), ptau_217_ (*R* ^2^_adj_. = 0.13), and FC of the cingulum (*R* ^2^_adj_. = 0.07), whereas NfL and FC of CC‐G were weaker (*R* ^2^_adj_. = 0.06), and GFAP was not significantly associated.

In the ADNI cohort, for executive functioning (ADNI‐EF2), the strongest associations were observed with amyloid‐PET and GFAP (both *R* ^2^_adj_. = 0.31), CC‐G FD (*R* ^2^_adj_. = 0.29), AD cortical thickness (*R* ^2^_adj_. = 0.27), and WMH volume (*R* ^2^_adj_. = 0.26) and FD of various fiber tracts (*R* ^2^_adj_. = 0.24–0.26).

**FIGURE 4:**
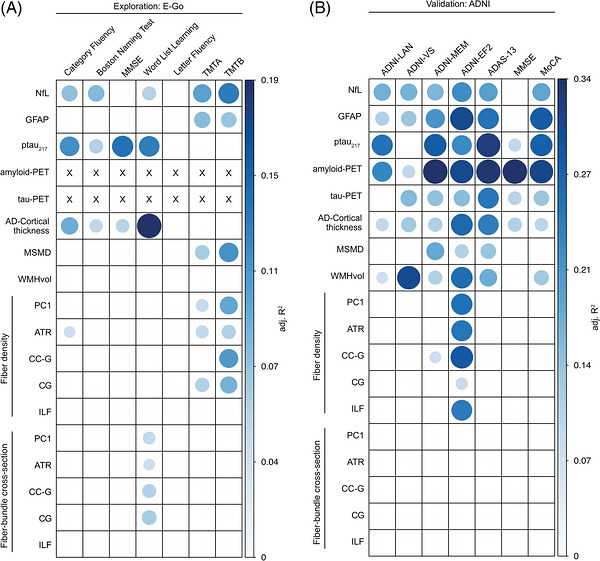
Associations between cognitive tests and plasma/ imaging biomarkers. Results from simple linear regression analyses obtained in (A) E‐Go and (B) ADNI. Effect sizes (adjusted R^2^) are represented by color (*p *< 0.05). ADAS‐13 = 13‐item Alzheimer's Disease Assessment Scale–Cognition; ADNI, Alzheimer's Disease Neuroimaging Initative; ADNI‐EF2, ADNI Executive Functioning 2 Composite Score; ADNI‐LAN, ADNI Language Composite Score; ADNI‐MEM, ADNI Memory Composite Score; ADNI‐VS, ADNI Visuospatial Functioning Composite Score; ATR, anterior thalamic radiation; CC‐G, genu of the corpus callosum; CG, cingulum; GFAP, glial fibrillary acidic protein; ILF, inferior longitudinal fasciculus; MMSE, Mini‐Mental State Examination; MoCA, Montreal Cognitive Assessment; MSMD, mean skeletonized mean diffusivity; NfL, neurofilament light chain; PC1, first principal component; ptau_217,_ phosphorylated tau _217_; TMTA/B, trial making test A/B, WMHvol, white matter hyperintensity volume (normalized to ICV).

For memory performance (ADNI‐MEM), representing a domain typically impaired in AD, the strongest associations were observed with amyloid‐PET (*R* ^2^_adj _= 0.34), ptau_217_ (*R* ^2^_adj_. = 0.29), and GFAP (*R* ^2^_adj _= 0.21), followed by MSMD (*R* ^2^_adj _= 0.18) and NfL (*R* ^2^_ad _= 0.16), while other markers such as FD‐PC1 and CC‐G FD contributed minimally. A full list of assessed variables can be found in Figure 4 and Table e‐S2.

**FIGURE 5:**
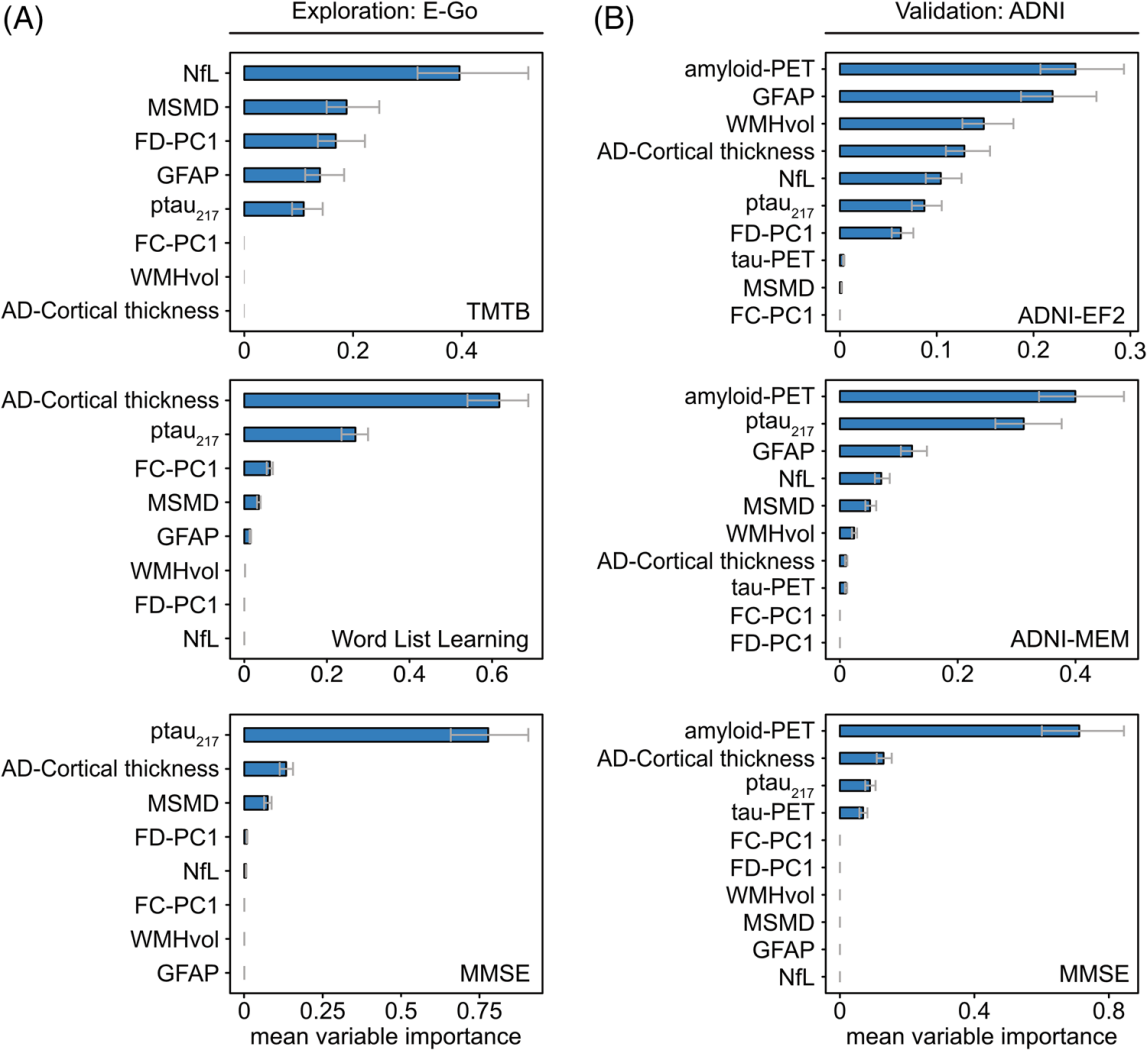
Random forest regression analyses showing the relative variable importance of various imaging and plasma markers for the respective cognitive test obtained in (A) E‐Go and (B) ADNI. Plots display point estimates with 95% confidence intervals for the conditional variable importance. ADNI, Alzheimer's Disease Neuroimaging Initiative; ADNI‐EF2, ADNI Executive Functioning 2 Composite Score; ADNI‐MEM, ADNI Memory Composite Score; FC‐PC1, first principal component of fiber‐bundle cross‐section; FD‐PC1, first principal component of fiber density; GFAP, glial fibrillary acidic protein; ICV, intracranial volume; MMSE, Mini‐Mental State Examination; MSMD, mean skeletonized mean diffusivity; NfL, neurofilament light chain; ptau_217,_ phosphorylated tau 217; TMTB, Trial Making Test (matrix B); WMHvol, white matter hyperintensity volume (normalized to ICV).

Random forest analyses confirmed these patterns (Figure 5). In the E‐Go cohort, random forest regression identified NfL (50.6%) and MSMD (30.1%) as the most important predictors of TMT‐B performance, whereas ptau_217_ (77.0%) and AD‐signature cortical thickness (27.5%) were the strongest predictors of Word List Learning performance.

In ADNI, we could validate our findings. For executive function (ADNI‐EF2), the strongest predictors were amyloid‐PET (24.3%), GFAP (22.0%), WMH volume (17.9%), AD‐signature cortical thickness (12.9%), NfL (12.6%), and FD PC1 (6.3%), whereas MSMD (0.2%) and ptau_217_ (8.7%) contributed less. For memory performance (ADNI‐MEM), global amyloid‐PET (40.0%) and ptau_217_ (31.2%) were the dominant predictors, followed by GFAP (12.2%), NfL (7.0%), MSMD (5.1%), WMH volume (2.4%), and AD‐signature cortical thickness (1.0%), with other markers contributing minimally.

Overall, across both cohorts, SVD‐typical processing speed/executive function deficits were primarily linked to NfL and SVD‐related diffusion markers, while AD‐typical episodic memory deficits were mainly associated with ptau_217_, amyloid‐PET, and AD‐signature cortical thickness. FD metrics captured SVD‐related deficits, while FC metrics reflected AD‐related neurodegeneration, confirming the domain‐specific relevance of these biomarkers.

## 4 DISCUSSION

In this study leveraging plasma biomarkers and advanced neuroimaging, we systematically evaluated the contributions of SVD‐ and AD‐related brain alterations to biomarker levels in a cross‐sectional sample of 76 memory clinic patients and validated our findings in an independent sample of 41 ADNI participants with additional gold‐standard amyloid‐ and tau‐PET. We also investigated the extent to which plasma and advanced fixel‐based biomarkers account for cognitive impairment characteristic of SVD and AD, respectively. Our findings indicate that NfL levels are most linked to SVD‐related white matter damage, while GFAP reflects both SVD‐ and AD‐related brain injury, with stronger associations with SVD. In contrast, ptau_217_ showed a robust and selective association with AD signature cortical thinning and amyloid‐ and tau‐PET, supporting its specificity for AD. Associations with cognition showed that NfL, GFAP, and FD were linked to deficits in processing speed and executive function, characteristic of SVD, whereas ptau_217_ and AD signature cortical thinning were most closely associated with episodic memory decline, typical of AD. Together, these results underscore the value of plasma biomarkers, particularly ptau_217_, in identifying cerebrovascular and neurodegenerative processes in patients with mixed pathology in memory clinic settings, and highlight the need to account for SVD contributions when interpreting NfL and GFAP levels in clinical contexts.

Our first finding revealed that NfL levels were primarily associated with imaging markers of SVD rather than with MRI indicators of AD‐related neurodegeneration. Previous research on familial SVD, that is, Cerebral Autosomal Dominant Arteriopathy with Subcortical Infarcts and Leukoencephalopathy (CADASIL), as well as sporadic SVD, already demonstrated that NfL levels are significantly elevated in SVD, and others found that increased NfL levels are associated with attenuated risk of long‐term mortality.^13^, ^57^ Our findings extend this earlier work by illustrating that, even in patients with potentially overlapping AD and SVD pathologies, SVD may emerge as the dominant factor elevating NfL levels. While it may seem unexpected that NfL levels were only to a small degree associated with AD signature cortical thinning in our exploration sample, this observation might be attributed to the relatively early stage of potential AD pathology in the E‐Go cohort. Specifically, participants were mostly CN and MCI, characterized by subjective or mildly detectable cognitive impairment, which represents a critical time window when individuals first notice symptoms and seek help in memory clinics. At these early stages, cortical atrophy is typically subtle, where tau pathology may not yet have produced significant axonal degeneration detectable by NfL.^58^ In contrast, SVD may cause diffuse white matter damage even in the absence of overt ischemic lesions, leading to chronic axonal disruption that may drive plasma NfL elevations.^2^, ^59^ Together, these findings underscore the importance of considering SVD as a major contributor to NfL elevations in clinical settings, particularly in patients without advanced AD‐related atrophy but with white matter hyperintensities, reduced FD, or other imaging features of SVD.

Our second major finding was that GFAP levels were associated with imaging markers of both SVD and AD. However, SVD imaging markers explained a substantially greater proportion of variance and dominated multivariable models. These results are consistent with a recent study in an Asian cohort, which found that the relationship between plasma GFAP and amyloid‐beta burden was moderated by cerebrovascular lesion load.^60^ Although our sample size did not allow for stratified analyses by ptau_217_ abnormality, our findings suggest that SVD may act as a key driver of GFAP elevation in memory clinic patients, particularly in early disease stages. This observation challenges emerging strategies that propose GFAP as a screening tool for Aβ positivity in clinical trials.^11^ If GFAP levels are significantly influenced by SVD, their utility as an enrichment marker for AD‐specific pathology may be limited, particularly in older populations where mixed pathologies are highly prevalent. Instead, GFAP may reflect broader neuroinflammatory activity at the intersection of AD and SVD processes. Supporting this, recent work in individuals with Down syndrome with both AD and cerebral amyloid angiopathy (CAA) pathology due to amyloid precursor protein (APP) gene triplication, demonstrated that cerebrovascular pathology may exacerbate astrocytosis and neurodegeneration even during presymptomatic stages.^61^ Further, the biological mechanisms behind plasma GFAP elevation remain incompletely understood. While astrocytosis is thought to release GFAP into the extracellular space, it is unclear how GFAP crosses into the bloodstream. Blood–brain barrier (BBB) dysfunction, commonly observed in SVD, may be a key contributor.^2^ Future multimodal studies incorporating BBB imaging techniques^62^ could help clarify the link between cerebrovascular damage and GFAP release into plasma, potentially refining its role as a fluid biomarker in mixed‐pathology populations.

Further, we found that ptau_217_ levels were robustly associated with imaging markers of AD and performance on episodic memory tests. This aligns with extensive prior research on ptau_217_ in amyloid‐positive cohorts,^63^, ^64^ and critically extends its specificity to mixed‐pathology populations. Even in the presence of co‐existing SVD, ptau_217_ remained the plasma marker most closely linked to core AD imaging markers and AD‐typical cognitive deficits. These findings highlight ptau_217_ as a highly promising candidate for routine diagnostic workups and clinical trial screening, particularly in identifying individuals at early AD stages.

Finally, when examining the relationship between fixel‐based metrics and cognition, we extended and validated our earlier findings:^16^ reduced FD, a marker of microstructural damage, was primarily associated with SVD‐typical cognitive impairment (i.e., slower processing speed and poorer executive function), while reductions in FC, reflecting macrostructural degeneration, were linked to AD‐typical episodic memory deficits in the E‐Go sample. Among the investigated tracts, the genu of the corpus callosum containing the forceps minor showed the strongest association with processing speed / executive function performance in both samples, supporting previous evidence of its strategic role in SVD‐related cognitive decline.^51^ These findings confirm that advanced diffusion MRI sensitively captures distinct patterns of white matter alterations that correspond to disease‐typical cognitive profiles associated with cerebrovascular and neurodegenerative brain alterations.

Our study has several limitations that need to be considered when interpreting our results. First, our samples sizes were relatively small, as datasets combining multi‐shell diffusion imaging with plasma biomarker measurements remain rare. Larger studies with harmonized multi‐shell acquisition protocols are therefore needed to validate and extend these findings. Second, full ATN classification was not available in our exploratory E‐Go cohort; however, we addressed this limitation by incorporating ADNI data, which, while modest in size, provided more complete biomarker characterization. Third, we did not have information on the presence of kidney disease available in our sample (e.g., glomerular filtration rate [GFR] levels). Prior studies have shown that reduced renal clearance can affect concentrations of markers such as NfL and ptau, potentially introducing unmeasured confounding.^65^ Finally, the two samples did not include fully harmonized neuropsychological test batteries, reflecting the independent design and data collection procedures of each study. While this methodological heterogeneity may be considered a limitation, it also allowed us to examine whether observed associations between biomarkers and cognition were consistent across samples despite differences in cognitive assessment, thereby supporting the generalizability of our findings.

In conclusion, our findings show that SVD is a major contributor to elevated plasma NfL and GFAP levels in memory clinic patients, with GFAP also being associated with AD. These results underscore the need to consider concomitant SVD when interpreting these biomarkers to avoid misattribution of SVD to AD‐related brain injury. Our data further support inflammation as a shared pathway of both SVD and AD. Extending previous work,^64^, ^66^ ptau_217_ emerged as the plasma marker most selectively aligned with AD‐related neurodegeneration and cognitive deficits, even in the presence of cerebrovascular comorbidity. From an imaging perspective, FD proved sensitive to SVD‐related microstructural changes and cognitive impairment. Together, our findings highlight the value of integrating plasma and imaging biomarkers to improve diagnostic accuracy in mixed‐pathology populations. Future studies should validate these findings in larger, longitudinal cohorts and incorporate additional modalities, such as BBB imaging and kidney function assessment, to refine biomarker interpretation in clinical practice.

## CONFLICTS OF INTEREST STATEMENT

The authors declare no conflicts of interest. Author disclosures are available in the Supporting Information.

## CONSENT STATEMENT

All study procedures complied with the Declaration of Helsinki. The E‐Go study received approval from the local ethics committee of the medical faculty of LMU Munich (project number 18‐930), and ethical approval for the ADNI study was obtained by the respective local ADNI investigators. Written informed consent was obtained from all participants.

## Supporting information



Supplementary Material: alz71530‐sup‐0001‐tablesS1‐S2.docx

Supplementary Material: alz71530‐sup‐0002‐SuppMat.pdf
