# Supplementary material for: Differential associations of plasma biomarkers with Alzheimer's disease and small vessel disease: A multimodal imaging study
Source: Alzheimers Dement. 2026 Jun 7;22(6):e71530. doi: 10.1002/alz.71530 (PMC13243208; doi:10.1002/alz.71530)
Supplement: Supplementary file 1 — Supplementary Material: alz71530‐sup‐0001‐tablesS1‐S2.docx [file ALZ-22-e71530-s001.docx]

**Supplementary Material.**

**Table e-1A)**: Simple linear regression models predicting plasma biomarker levels (adjusted R^2^, *p* < 0.05) in E-Go.

| Plasma biomarker | Predictor | Std. β | SE | CI lower | CI upper | *p* | Adj. R^2^ |
| --- | --- | --- | --- | --- | --- | --- | --- |
| NfL | AD-Cortical Thickness | -0.261 | 0.112 | -0.485 | -0.038 | 0.0226 | 0.056 |
| NfL | MSMD | 0.467 | 0.103 | 0.262 | 0.672 | <0.001 | 0.207 |
| NfL | WMHvol | 0.386 | 0.107 | 0.172 | 0.599 | <0.001 | 0.137 |
| NfL | AF-FD | -0.424 | 0.105 | -0.634 | -0.215 | <0.001 | 0.169 |
| NfL | UF-FD | -0.367 | 0.108 | -0.582 | -0.152 | 0.00111 | 0.123 |
| NfL | IFO-FD | -0.461 | 0.103 | -0.667 | -0.256 | <0.001 | 0.202 |
| NfL | MLF-FD | -0.296 | 0.111 | -0.517 | -0.075 | 0.00946 | 0.075 |
| NfL | ILF-FD | -0.337 | 0.109 | -0.555 | -0.119 | 0.00293 | 0.101 |
| NfL | SLF_I-FD | -0.331 | 0.11 | -0.549 | -0.112 | 0.00354 | 0.097 |
| NfL | SLF_II-FD | -0.344 | 0.109 | -0.561 | -0.126 | 0.00237 | 0.106 |
| NfL | SLF_III-FD | -0.388 | 0.107 | -0.601 | -0.174 | <0.001 | 0.139 |
| NfL | T_PREF-FD | -0.446 | 0.104 | -0.653 | -0.238 | <0.001 | 0.188 |
| NfL | T_PREM-FD | -0.323 | 0.11 | -0.542 | -0.103 | 0.00449 | 0.092 |
| NfL | T_PREC-FD | - | - | - | - | - | - |
| NfL | T_POSTC-FD | - | - | - | - | - | - |
| NfL | T_PAR-FD | - | - | - | - | - | - |
| NfL | T_OCC-FD | -0.335 | 0.11 | -0.554 | -0.117 | 0.00306 | 0.101 |
| NfL | ATR-FD | -0.446 | 0.104 | -0.653 | -0.238 | <0.001 | 0.188 |
| NfL | STR-FD | - | - | - | - | - | - |
| NfL | OR-FD | -0.334 | 0.11 | -0.552 | -0.116 | 0.0032 | 0.1 |
| NfL | FPT-FD | -0.331 | 0.11 | -0.55 | -0.113 | 0.00347 | 0.098 |
| NfL | CST-FD | - | - | - | - | - | - |
| NfL | POPT-FD | - | - | - | - | - | - |
| NfL | CC_1-FD | -0.376 | 0.108 | -0.591 | -0.162 | <0.001 | 0.13 |
| NfL | CC-G-FD | -0.468 | 0.103 | -0.673 | -0.263 | <0.001 | 0.208 |
| NfL | CC_3-FD | -0.382 | 0.107 | -0.596 | -0.168 | <0.001 | 0.135 |
| NfL | CC_4-FD | -0.269 | 0.112 | -0.492 | -0.046 | 0.0186 | 0.06 |
| NfL | CC_5-FD | -0.275 | 0.112 | -0.498 | -0.052 | 0.0161 | 0.063 |
| NfL | CC_6-FD | - | - | - | - | - | - |
| NfL | CC_7-FD | -0.27 | 0.112 | -0.493 | -0.047 | 0.0181 | 0.061 |
| NfL | CA-FD | -0.36 | 0.108 | -0.576 | -0.144 | 0.00141 | 0.118 |
| NfL | CG-FD | -0.373 | 0.108 | -0.588 | -0.158 | <0.001 | 0.127 |
| NfL | AF-FC | - | - | - | - | - | - |
| NfL | UF-FC | -0.226 | 0.113 | -0.452 | -0.001 | 0.0492 | 0.038 |
| NfL | IFO-FC | - | - | - | - | - | - |
| NfL | MLF-FC | - | - | - | - | - | - |
| NfL | ILF-FC | - | - | - | - | - | - |
| NfL | SLF_I-FC | - | - | - | - | - | - |
| NfL | SLF_II-FC | - | - | - | - | - | - |
| NfL | SLF_III-FC | - | - | - | - | - | - |
| NfL | T_PREF-FC | - | - | - | - | - | - |
| NfL | T_PREM-FC | - | - | - | - | - | - |
| NfL | T_PREC-FC | - | - | - | - | - | - |
| NfL | T_POSTC-FC | - | - | - | - | - | - |
| NfL | T_PAR-FC | - | - | - | - | - | - |
| NfL | T_OCC-FC | - | - | - | - | - | - |
| NfL | ATR-FC | - | - | - | - | - | - |
| NfL | STR-FC | -0.229 | 0.113 | -0.454 | -0.003 | 0.0469 | 0.039 |
| NfL | OR-FC | - | - | - | - | - | - |
| NfL | FPT-FC | - | - | - | - | - | - |
| NfL | CST-FC | - | - | - | - | - | - |
| NfL | POPT-FC | - | - | - | - | - | - |
| NfL | CC_1-FC | - | - | - | - | - | - |
| NfL | CC-G-FC | - | - | - | - | - | - |
| NfL | CC_3-FC | - | - | - | - | - | - |
| NfL | CC_4-FC | - | - | - | - | - | - |
| NfL | CC_5-FC | - | - | - | - | - | - |
| NfL | CC_6-FC | - | - | - | - | - | - |
| NfL | CC_7-FC | - | - | - | - | - | - |
| NfL | CA-FC | -0.316 | 0.11 | -0.536 | -0.096 | 0.0054 | 0.088 |
| NfL | CG-FC | - | - | - | - | - | - |
| GFAP | AD-Cortical Thickness | -0.428 | 0.105 | -0.637 | -0.219 | <0.001 | 0.172 |
| GFAP | MSMD | 0.434 | 0.105 | 0.225 | 0.643 | <0.001 | 0.177 |
| GFAP | WMHvol | 0.41 | 0.106 | 0.199 | 0.622 | <0.001 | 0.157 |
| GFAP | AF-FD | -0.377 | 0.108 | -0.592 | -0.163 | <0.001 | 0.131 |
| GFAP | UF-FD | -0.423 | 0.105 | -0.633 | -0.213 | <0.001 | 0.168 |
| GFAP | IFO-FD | -0.52 | 0.099 | -0.718 | -0.323 | <0.001 | 0.261 |
| GFAP | MLF-FD | -0.404 | 0.106 | -0.616 | -0.192 | <0.001 | 0.152 |
| GFAP | ILF-FD | -0.39 | 0.107 | -0.603 | -0.177 | <0.001 | 0.141 |
| GFAP | SLF_I-FD | -0.267 | 0.112 | -0.49 | -0.044 | 0.0197 | 0.059 |
| GFAP | SLF_II-FD | -0.279 | 0.112 | -0.502 | -0.057 | 0.0146 | 0.065 |
| GFAP | SLF_III-FD | -0.344 | 0.109 | -0.561 | -0.126 | 0.00235 | 0.106 |
| GFAP | T_PREF-FD | -0.32 | 0.11 | -0.54 | -0.101 | 0.0048 | 0.09 |
| GFAP | T_PREM-FD | - | - | - | - | - | - |
| GFAP | T_PREC-FD | - | - | - | - | - | - |
| GFAP | T_POSTC-FD | - | - | - | - | - | - |
| GFAP | T_PAR-FD | -0.284 | 0.111 | -0.506 | -0.062 | 0.0129 | 0.068 |
| GFAP | T_OCC-FD | -0.418 | 0.106 | -0.629 | -0.208 | <0.001 | 0.164 |
| GFAP | ATR-FD | -0.385 | 0.107 | -0.599 | -0.172 | <0.001 | 0.137 |
| GFAP | STR-FD | - | - | - | - | - | - |
| GFAP | OR-FD | -0.424 | 0.105 | -0.634 | -0.214 | <0.001 | 0.169 |
| GFAP | FPT-FD | - | - | - | - | - | - |
| GFAP | CST-FD | - | - | - | - | - | - |
| GFAP | POPT-FD | - | - | - | - | - | - |
| GFAP | CC_1-FD | -0.45 | 0.104 | -0.657 | -0.243 | <0.001 | 0.192 |
| GFAP | CC-G-FD | -0.439 | 0.104 | -0.647 | -0.231 | <0.001 | 0.182 |
| GFAP | CC_3-FD | -0.353 | 0.109 | -0.57 | -0.136 | 0.00176 | 0.113 |
| GFAP | CC_4-FD | -0.366 | 0.108 | -0.581 | -0.15 | 0.00116 | 0.122 |
| GFAP | CC_5-FD | -0.359 | 0.108 | -0.576 | -0.143 | 0.00143 | 0.117 |
| GFAP | CC_6-FD | -0.366 | 0.108 | -0.582 | -0.151 | 0.00114 | 0.122 |
| GFAP | CC_7-FD | -0.425 | 0.105 | -0.635 | -0.215 | <0.001 | 0.17 |
| GFAP | CA-FD | -0.331 | 0.11 | -0.55 | -0.113 | 0.00346 | 0.098 |
| GFAP | CG-FD | -0.435 | 0.105 | -0.643 | -0.226 | <0.001 | 0.178 |
| GFAP | AF-FC | - | - | - | - | - | - |
| GFAP | UF-FC | - | - | - | - | - | - |
| GFAP | IFO-FC | - | - | - | - | - | - |
| GFAP | MLF-FC | - | - | - | - | - | - |
| GFAP | ILF-FC | - | - | - | - | - | - |
| GFAP | SLF_I-FC | - | - | - | - | - | - |
| GFAP | SLF_II-FC | - | - | - | - | - | - |
| GFAP | SLF_III-FC | - | - | - | - | - | - |
| GFAP | T_PREF-FC | - | - | - | - | - | - |
| GFAP | T_PREM-FC | - | - | - | - | - | - |
| GFAP | T_PREC-FC | - | - | - | - | - | - |
| GFAP | T_POSTC-FC | - | - | - | - | - | - |
| GFAP | T_PAR-FC | - | - | - | - | - | - |
| GFAP | T_OCC-FC | - | - | - | - | - | - |
| GFAP | ATR-FC | - | - | - | - | - | - |
| GFAP | STR-FC | - | - | - | - | - | - |
| GFAP | OR-FC | - | - | - | - | - | - |
| GFAP | FPT-FC | - | - | - | - | - | - |
| GFAP | CST-FC | - | - | - | - | - | - |
| GFAP | POPT-FC | - | - | - | - | - | - |
| GFAP | CC_1-FC | - | - | - | - | - | - |
| GFAP | CC-G-FC | - | - | - | - | - | - |
| GFAP | CC_3-FC | - | - | - | - | - | - |
| GFAP | CC_4-FC | - | - | - | - | - | - |
| GFAP | CC_5-FC | - | - | - | - | - | - |
| GFAP | CC_6-FC | - | - | - | - | - | - |
| GFAP | CC_7-FC | - | - | - | - | - | - |
| GFAP | CA-FC | - | - | - | - | - | - |
| GFAP | CG-FC | - | - | - | - | - | - |
| Ptau_217_ | AD-Cortical Thickness | -0.471 | 0.103 | -0.675 | -0.266 | <0.001 | 0.211 |
| Ptau_217_ | MSMD | 0.25 | 0.113 | 0.025 | 0.474 | 0.0297 | 0.05 |
| Ptau_217_ | WMHvol | - | - | - | - | - | - |
| Ptau_217_ | AF-FD | -0.253 | 0.112 | -0.477 | -0.029 | 0.0275 | 0.051 |
| Ptau_217_ | UF-FD | - | - | - | - | - | - |
| Ptau_217_ | IFO-FD | -0.288 | 0.111 | -0.51 | -0.066 | 0.0117 | 0.071 |
| Ptau_217_ | MLF-FD | -0.373 | 0.108 | -0.588 | -0.158 | <0.001 | 0.128 |
| Ptau_217_ | ILF-FD | - | - | - | - | - | - |
| Ptau_217_ | SLF_I-FD | - | - | - | - | - | - |
| Ptau_217_ | SLF_II-FD | - | - | - | - | - | - |
| Ptau_217_ | SLF_III-FD | -0.313 | 0.11 | -0.533 | -0.093 | 0.00587 | 0.086 |
| Ptau_217_ | T_PREF-FD | - | - | - | - | - | - |
| Ptau_217_ | T_PREM-FD | - | - | - | - | - | - |
| Ptau_217_ | T_PREC-FD | - | - | - | - | - | - |
| Ptau_217_ | T_POSTC-FD | - | - | - | - | - | - |
| Ptau_217_ | T_PAR-FD | -0.328 | 0.11 | -0.547 | -0.11 | 0.00377 | 0.096 |
| Ptau_217_ | T_OCC-FD | -0.326 | 0.11 | -0.545 | -0.107 | 0.0041 | 0.094 |
| Ptau_217_ | ATR-FD | - | - | - | - | - | - |
| Ptau_217_ | STR-FD | - | - | - | - | - | - |
| Ptau_217_ | OR-FD | -0.327 | 0.11 | -0.546 | -0.108 | 0.0039 | 0.095 |
| Ptau_217_ | FPT-FD | - | - | - | - | - | - |
| Ptau_217_ | CST-FD | - | - | - | - | - | - |
| Ptau_217_ | POPT-FD | -0.326 | 0.11 | -0.545 | -0.107 | 0.00408 | 0.094 |
| Ptau_217_ | CC_1-FD | -0.278 | 0.112 | -0.501 | -0.056 | 0.0149 | 0.065 |
| Ptau_217_ | CC-G-FD | -0.237 | 0.113 | -0.462 | -0.012 | 0.0392 | 0.043 |
| Ptau_217_ | CC_3-FD | - | - | - | - | - | - |
| Ptau_217_ | CC_4-FD | - | - | - | - | - | - |
| Ptau_217_ | CC_5-FD | -0.262 | 0.112 | -0.486 | -0.039 | 0.0222 | 0.056 |
| Ptau_217_ | CC_6-FD | -0.381 | 0.107 | -0.595 | -0.167 | <0.001 | 0.134 |
| Ptau_217_ | CC_7-FD | -0.322 | 0.11 | -0.541 | -0.102 | 0.00462 | 0.091 |
| Ptau_217_ | CA-FD | -0.29 | 0.111 | -0.512 | -0.069 | 0.0109 | 0.072 |
| Ptau_217_ | CG-FD | -0.252 | 0.113 | -0.476 | -0.028 | 0.0282 | 0.051 |
| Ptau_217_ | AF-FC | - | - | - | - | - | - |
| Ptau_217_ | UF-FC | - | - | - | - | - | - |
| Ptau_217_ | IFO-FC | - | - | - | - | - | - |
| Ptau_217_ | MLF-FC | - | - | - | - | - | - |
| Ptau_217_ | ILF-FC | - | - | - | - | - | - |
| Ptau_217_ | SLF_I-FC | - | - | - | - | - | - |
| Ptau_217_ | SLF_II-FC | - | - | - | - | - | - |
| Ptau_217_ | SLF_III-FC | - | - | - | - | - | - |
| Ptau_217_ | T_PREF-FC | - | - | - | - | - | - |
| Ptau_217_ | T_PREM-FC | - | - | - | - | - | - |
| Ptau_217_ | T_PREC-FC | - | - | - | - | - | - |
| Ptau_217_ | T_POSTC-FC | - | - | - | - | - | - |
| Ptau_217_ | T_PAR-FC | - | - | - | - | - | - |
| Ptau_217_ | T_OCC-FC | 0.292 | 0.111 | 0.07 | 0.513 | 0.0105 | 0.073 |
| Ptau_217_ | ATR-FC | - | - | - | - | - | - |
| Ptau_217_ | STR-FC | - | - | - | - | - | - |
| Ptau_217_ | OR-FC | 0.29 | 0.111 | 0.069 | 0.512 | 0.011 | 0.072 |
| Ptau_217_ | FPT-FC | - | - | - | - | - | - |
| Ptau_217_ | CST-FC | - | - | - | - | - | - |
| Ptau_217_ | POPT-FC | - | - | - | - | - | - |
| Ptau_217_ | CC_1-FC | - | - | - | - | - | - |
| Ptau_217_ | CC-G-FC | - | - | - | - | - | - |
| Ptau_217_ | CC_3-FC | - | - | - | - | - | - |
| Ptau_217_ | CC_4-FC | - | - | - | - | - | - |
| Ptau_217_ | CC_5-FC | - | - | - | - | - | - |
| Ptau_217_ | CC_6-FC | - | - | - | - | - | - |
| Ptau_217_ | CC_7-FC | 0.276 | 0.112 | 0.054 | 0.499 | 0.0157 | 0.064 |
| Ptau_217_ | CA-FC | - | - | - | - | - | - |
| Ptau_217_ | CG-FC | - | - | - | - | - | - |

**Table e-1B)**: Simple linear regression models predicting plasma biomarker levels (adjusted R^2^, *p* < 0.05) in ADNI.

| **Plasma biomarker** | **Predictor** | **Std. β** | **SE** | **CI lower** | **CI upper** | ***p*** | **Adj. R^2^** |
| --- | --- | --- | --- | --- | --- | --- | --- |
| NfL | amyloid-PET | 0.378 | 0.148 | 0.078 | 0.678 | 0.0149 | 0.121 |
| NfL | tau-PET | - | - | - | - | - | - |
| NfL | AD-Cortical Thickness | -0.531 | 0.136 | -0.806 | -0.257 | <0.001 | 0.264 |
| NfL | MSMD | 0.42 | 0.145 | 0.126 | 0.714 | 0.00627 | 0.155 |
| NfL | WMHvol | 0.544 | 0.134 | 0.272 | 0.816 | <0.001 | 0.278 |
| NfL | AF-FD | - | - | - | - | - | - |
| NfL | UF-FD | - | - | - | - | - | - |
| NfL | IFO-FD | -0.345 | 0.15 | -0.649 | -0.041 | 0.0273 | 0.096 |
| NfL | MLF-FD | - | - | - | - | - | - |
| NfL | ILF-FD | - | - | - | - | - | - |
| NfL | SLF-I-FD | - | - | - | - | - | - |
| NfL | SLF-II-FD | -0.352 | 0.15 | -0.655 | -0.048 | 0.0242 | 0.101 |
| NfL | SLF-III-FD | -0.309 | 0.152 | -0.617 | 0 | 0.0497 | 0.072 |
| NfL | T-PREF-FD | -0.413 | 0.146 | -0.708 | -0.119 | 0.00721 | 0.15 |
| NfL | T-PREM-FD | - | - | - | - | - | - |
| NfL | T-PREC-FD | -0.34 | 0.151 | -0.644 | -0.035 | 0.0298 | 0.093 |
| NfL | T-POSTC-FD | - | - | - | - | - | - |
| NfL | T-PAR-FD | - | - | - | - | - | - |
| NfL | T-OCC-FD | - | - | - | - | - | - |
| NfL | ATR-FD | -0.357 | 0.15 | -0.66 | -0.055 | 0.0218 | 0.105 |
| NfL | STR-FD | - | - | - | - | - | - |
| NfL | OR-FD | - | - | - | - | - | - |
| NfL | FPT-FD | - | - | - | - | - | - |
| NfL | CST-FD | - | - | - | - | - | - |
| NfL | POPT-FD | - | - | - | - | - | - |
| NfL | CC-1-FD | -0.381 | 0.148 | -0.681 | -0.082 | 0.0139 | 0.124 |
| NfL | CC-G-FD | -0.48 | 0.14 | -0.764 | -0.196 | 0.00149 | 0.211 |
| NfL | CC-3-FD | -0.423 | 0.145 | -0.717 | -0.13 | 0.00582 | 0.158 |
| NfL | CC-4-FD | -0.502 | 0.139 | -0.782 | -0.221 | <0.001 | 0.232 |
| NfL | CC-5-FD | -0.386 | 0.148 | -0.685 | -0.088 | 0.0126 | 0.128 |
| NfL | CC-6-FD | - | - | - | - | - | - |
| NfL | CC-7-FD | - | - | - | - | - | - |
| NfL | CA-FD | - | - | - | - | - | - |
| NfL | CG-FD | -0.423 | 0.145 | -0.717 | -0.13 | 0.00585 | 0.158 |
| NfL | AF-FC | - | - | - | - | - | - |
| NfL | UF-FC | - | - | - | - | - | - |
| NfL | IFO-FC | - | - | - | - | - | - |
| NfL | MLF-FC | - | - | - | - | - | - |
| NfL | ILF-FC | - | - | - | - | - | - |
| NfL | SLF-I-FC | - | - | - | - | - | - |
| NfL | SLF-II-FC | - | - | - | - | - | - |
| NfL | SLF-III-FC | - | - | - | - | - | - |
| NfL | T-PREF-FC | - | - | - | - | - | - |
| NfL | T-PREM-FC | - | - | - | - | - | - |
| NfL | T-PREC-FC | - | - | - | - | - | - |
| NfL | T-POSTC-FC | - | - | - | - | - | - |
| NfL | T-PAR-FC | - | - | - | - | - | - |
| NfL | T-OCC-FC | - | - | - | - | - | - |
| NfL | ATR-FC | - | - | - | - | - | - |
| NfL | STR-FC | - | - | - | - | - | - |
| NfL | OR-FC | - | - | - | - | - | - |
| NfL | FPT-FC | - | - | - | - | - | - |
| NfL | CST-FC | - | - | - | - | - | - |
| NfL | POPT-FC | - | - | - | - | - | - |
| NfL | CC-1-FC | - | - | - | - | - | - |
| NfL | CC-G-FC | - | - | - | - | - | - |
| NfL | CC-3-FC | - | - | - | - | - | - |
| NfL | CC-4-FC | - | - | - | - | - | - |
| NfL | CC-5-FC | - | - | - | - | - | - |
| NfL | CC-6-FC | - | - | - | - | - | - |
| NfL | CC-7-FC | - | - | - | - | - | - |
| NfL | CA-FC | - | - | - | - | - | - |
| NfL | CG-FC | - | - | - | - | - | - |
| GFAP | amyloid-PET | 0.472 | 0.141 | 0.187 | 0.758 | 0.00182 | 0.203 |
| GFAP | tau-PET | 0.343 | 0.15 | 0.039 | 0.647 | 0.0281 | 0.095 |
| GFAP | AD-Cortical Thickness | -0.47 | 0.141 | -0.756 | -0.184 | 0.00194 | 0.201 |
| GFAP | MSMD | 0.461 | 0.142 | 0.174 | 0.749 | 0.00241 | 0.192 |
| GFAP | WMHvol | 0.38 | 0.148 | 0.081 | 0.68 | 0.0141 | 0.123 |
| GFAP | AF-FD | -0.474 | 0.141 | -0.759 | -0.189 | 0.00173 | 0.205 |
| GFAP | UF-FD | - | - | - | - | - | - |
| GFAP | IFO-FD | -0.443 | 0.144 | -0.733 | -0.152 | 0.00375 | 0.175 |
| GFAP | MLF-FD | -0.308 | 0.152 | -0.616 | 0 | 0.0499 | 0.072 |
| GFAP | ILF-FD | -0.394 | 0.147 | -0.691 | -0.096 | 0.0109 | 0.133 |
| GFAP | SLF-I-FD | - | - | - | - | - | - |
| GFAP | SLF-II-FD | -0.364 | 0.149 | -0.665 | -0.062 | 0.0194 | 0.11 |
| GFAP | SLF-III-FD | -0.453 | 0.143 | -0.742 | -0.164 | 0.00294 | 0.185 |
| GFAP | T-PREF-FD | -0.447 | 0.143 | -0.736 | -0.157 | 0.00342 | 0.179 |
| GFAP | T-PREM-FD | -0.399 | 0.147 | -0.696 | -0.103 | 0.00967 | 0.138 |
| GFAP | T-PREC-FD | -0.403 | 0.147 | -0.699 | -0.106 | 0.00905 | 0.141 |
| GFAP | T-POSTC-FD | -0.368 | 0.149 | -0.669 | -0.067 | 0.0178 | 0.113 |
| GFAP | T-PAR-FD | - | - | - | - | - | - |
| GFAP | T-OCC-FD | -0.407 | 0.146 | -0.703 | -0.111 | 0.00823 | 0.144 |
| GFAP | ATR-FD | -0.401 | 0.147 | -0.698 | -0.104 | 0.00939 | 0.139 |
| GFAP | STR-FD | -0.333 | 0.151 | -0.638 | -0.027 | 0.0335 | 0.088 |
| GFAP | OR-FD | -0.405 | 0.146 | -0.701 | -0.109 | 0.00856 | 0.143 |
| GFAP | FPT-FD | -0.402 | 0.147 | -0.698 | -0.105 | 0.0092 | 0.14 |
| GFAP | CST-FD | -0.394 | 0.147 | -0.692 | -0.096 | 0.0108 | 0.133 |
| GFAP | POPT-FD | - | - | - | - | - | - |
| GFAP | CC-1-FD | -0.428 | 0.145 | -0.721 | -0.135 | 0.00528 | 0.162 |
| GFAP | CC-G-FD | -0.389 | 0.148 | -0.687 | -0.091 | 0.0119 | 0.13 |
| GFAP | CC-3-FD | -0.399 | 0.147 | -0.696 | -0.102 | 0.00971 | 0.138 |
| GFAP | CC-4-FD | -0.411 | 0.146 | -0.707 | -0.116 | 0.00755 | 0.148 |
| GFAP | CC-5-FD | -0.319 | 0.152 | -0.626 | -0.012 | 0.0419 | 0.079 |
| GFAP | CC-6-FD | -0.339 | 0.151 | -0.644 | -0.035 | 0.03 | 0.092 |
| GFAP | CC-7-FD | -0.361 | 0.149 | -0.663 | -0.059 | 0.0204 | 0.108 |
| GFAP | CA-FD | -0.337 | 0.151 | -0.642 | -0.032 | 0.0312 | 0.091 |
| GFAP | CG-FD | -0.429 | 0.145 | -0.722 | -0.137 | 0.00512 | 0.163 |
| GFAP | AF-FC | -0.318 | 0.152 | -0.625 | -0.011 | 0.0425 | 0.078 |
| GFAP | UF-FC | -0.401 | 0.147 | -0.698 | -0.105 | 0.0093 | 0.14 |
| GFAP | IFO-FC | - | - | - | - | - | - |
| GFAP | MLF-FC | -0.34 | 0.151 | -0.645 | -0.036 | 0.0294 | 0.093 |
| GFAP | ILF-FC | -0.373 | 0.149 | -0.673 | -0.072 | 0.0164 | 0.117 |
| GFAP | SLF-I-FC | - | - | - | - | - | - |
| GFAP | SLF-II-FC | - | - | - | - | - | - |
| GFAP | SLF-III-FC | - | - | - | - | - | - |
| GFAP | T-PREF-FC | - | - | - | - | - | - |
| GFAP | T-PREM-FC | - | - | - | - | - | - |
| GFAP | T-PREC-FC | - | - | - | - | - | - |
| GFAP | T-POSTC-FC | -0.343 | 0.15 | -0.647 | -0.039 | 0.0281 | 0.095 |
| GFAP | T-PAR-FC | - | - | - | - | - | - |
| GFAP | T-OCC-FC | - | - | - | - | - | - |
| GFAP | ATR-FC | - | - | - | - | - | - |
| GFAP | STR-FC | - | - | - | - | - | - |
| GFAP | OR-FC | - | - | - | - | - | - |
| GFAP | FPT-FC | - | - | - | - | - | - |
| GFAP | CST-FC | - | - | - | - | - | - |
| GFAP | POPT-FC | - | - | - | - | - | - |
| GFAP | CC-1-FC | - | - | - | - | - | - |
| GFAP | CC-G-FC | - | - | - | - | - | - |
| GFAP | CC-3-FC | - | - | - | - | - | - |
| GFAP | CC-4-FC | - | - | - | - | - | - |
| GFAP | CC-5-FC | - | - | - | - | - | - |
| GFAP | CC-6-FC | -0.321 | 0.152 | -0.628 | -0.014 | 0.0408 | 0.08 |
| GFAP | CC-7-FC | - | - | - | - | - | - |
| GFAP | CA-FC | -0.455 | 0.143 | -0.743 | -0.166 | 0.00281 | 0.187 |
| GFAP | CG-FC | - | - | - | - | - | - |
| Ptau_217_ | amyloid-PET | 0.611 | 0.127 | 0.354 | 0.867 | <0.001 | 0.357 |
| Ptau_217_ | tau-PET | 0.74 | 0.108 | 0.522 | 0.958 | <0.001 | 0.535 |
| Ptau_217_ | AD-Cortical Thickness | -0.543 | 0.134 | -0.815 | -0.271 | <0.001 | 0.276 |
| Ptau_217_ | MSMD | 0.392 | 0.147 | 0.095 | 0.69 | 0.0112 | 0.132 |
| Ptau_217_ | WMHvol | - | - | - | - | - | - |
| Ptau_217_ | AF-FD | - | - | - | - | - | - |
| Ptau_217_ | UF-FD | - | - | - | - | - | - |
| Ptau_217_ | IFO-FD | - | - | - | - | - | - |
| Ptau_217_ | MLF-FD | - | - | - | - | - | - |
| Ptau_217_ | ILF-FD | - | - | - | - | - | - |
| Ptau_217_ | SLF-I-FD | - | - | - | - | - | - |
| Ptau_217_ | SLF-II-FD | -0.32 | 0.152 | -0.627 | -0.013 | 0.0414 | 0.079 |
| Ptau_217_ | SLF-III-FD | - | - | - | - | - | - |
| Ptau_217_ | T-PREF-FD | - | - | - | - | - | - |
| Ptau_217_ | T-PREM-FD | - | - | - | - | - | - |
| Ptau_217_ | T-PREC-FD | - | - | - | - | - | - |
| Ptau_217_ | T-POSTC-FD | - | - | - | - | - | - |
| Ptau_217_ | T-PAR-FD | - | - | - | - | - | - |
| Ptau_217_ | T-OCC-FD | - | - | - | - | - | - |
| Ptau_217_ | ATR-FD | -0.329 | 0.151 | -0.635 | -0.023 | 0.0358 | 0.085 |
| Ptau_217_ | STR-FD | - | - | - | - | - | - |
| Ptau_217_ | OR-FD | - | - | - | - | - | - |
| Ptau_217_ | FPT-FD | - | - | - | - | - | - |
| Ptau_217_ | CST-FD | - | - | - | - | - | - |
| Ptau_217_ | POPT-FD | - | - | - | - | - | - |
| Ptau_217_ | CC-1-FD | - | - | - | - | - | - |
| Ptau_217_ | CC-G-FD | - | - | - | - | - | - |
| Ptau_217_ | CC-3-FD | - | - | - | - | - | - |
| Ptau_217_ | CC-4-FD | - | - | - | - | - | - |
| Ptau_217_ | CC-5-FD | - | - | - | - | - | - |
| Ptau_217_ | CC-6-FD | - | - | - | - | - | - |
| Ptau_217_ | CC-7-FD | - | - | - | - | - | - |
| Ptau_217_ | CA-FD | - | - | - | - | - | - |
| Ptau_217_ | CG-FD | - | - | - | - | - | - |
| Ptau_217_ | AF-FC | - | - | - | - | - | - |
| Ptau_217_ | UF-FC | -0.332 | 0.151 | -0.638 | -0.027 | 0.0337 | 0.088 |
| Ptau_217_ | IFO-FC | - | - | - | - | - | - |
| Ptau_217_ | MLF-FC | - | - | - | - | - | - |
| Ptau_217_ | ILF-FC | - | - | - | - | - | - |
| Ptau_217_ | SLF-I-FC | - | - | - | - | - | - |
| Ptau_217_ | SLF-II-FC | - | - | - | - | - | - |
| Ptau_217_ | SLF-III-FC | - | - | - | - | - | - |
| Ptau_217_ | T-PREF-FC | - | - | - | - | - | - |
| Ptau_217_ | T-PREM-FC | - | - | - | - | - | - |
| Ptau_217_ | T-PREC-FC | -0.342 | 0.15 | -0.646 | -0.037 | 0.0288 | 0.094 |
| Ptau_217_ | T-POSTC-FC | - | - | - | - | - | - |
| Ptau_217_ | T-PAR-FC | - | - | - | - | - | - |
| Ptau_217_ | T-OCC-FC | - | - | - | - | - | - |
| Ptau_217_ | ATR-FC | - | - | - | - | - | - |
| Ptau_217_ | STR-FC | - | - | - | - | - | - |
| Ptau_217_ | OR-FC | - | - | - | - | - | - |
| Ptau_217_ | FPT-FC | - | - | - | - | - | - |
| Ptau_217_ | CST-FC | - | - | - | - | - | - |
| Ptau_217_ | POPT-FC | - | - | - | - | - | - |
| Ptau_217_ | CC-1-FC | - | - | - | - | - | - |
| Ptau_217_ | CC-G-FC | - | - | - | - | - | - |
| Ptau_217_ | CC-3-FC | - | - | - | - | - | - |
| Ptau_217_ | CC-4-FC | - | - | - | - | - | - |
| Ptau_217_ | CC-5-FC | - | - | - | - | - | - |
| Ptau_217_ | CC-6-FC | - | - | - | - | - | - |
| Ptau_217_ | CC-7-FC | - | - | - | - | - | - |
| Ptau_217_ | CA-FC | - | - | - | - | - | - |
| Ptau_217_ | CG-FC | - | - | - | - | - | - |

**Table e-2A**: Simple linear regression models predicting neuropsychological performance (adjusted R^2^, *p* < 0.05) in E-Go.

| **Cognition** | **Predictor** | **Std. β** | **SE** | **CI lower** | **CI upper** | ***p*** | **Adj. R^2^** |
| --- | --- | --- | --- | --- | --- | --- | --- |
| Category Fluency | NfL | -0.299 | 0.111 | -0.52 | -0.078 | 0.00866 | 0.077 |
| Category Fluency | GFAP | 0 | 0 | 0 | 0 | 0 | 0 |
| Category Fluency | Ptau217 | -0.36 | 0.108 | -0.576 | -0.144 | 0.0014 | 0.118 |
| Category Fluency | AD-Cortical Thickness | 0.323 | 0.11 | 0.104 | 0.542 | 0.00446 | 0.092 |
| Category Fluency | MSMD | 0 | 0 | 0 | 0 | 0 | 0 |
| Category Fluency | WMHvol | 0 | 0 | 0 | 0 | 0 | 0 |
| Category Fluency | AF-FD | 0 | 0 | 0 | 0 | 0 | 0 |
| Category Fluency | UF-FD | 0 | 0 | 0 | 0 | 0 | 0 |
| Category Fluency | IFO-FD | 0 | 0 | 0 | 0 | 0 | 0 |
| Category Fluency | MLF-FD | 0 | 0 | 0 | 0 | 0 | 0 |
| Category Fluency | ILF-FD | 0 | 0 | 0 | 0 | 0 | 0 |
| Category Fluency | SLF-I-FD | 0 | 0 | 0 | 0 | 0 | 0 |
| Category Fluency | SLF-II-FD | 0 | 0 | 0 | 0 | 0 | 0 |
| Category Fluency | SLF-III-FD | 0.25 | 0.113 | 0.026 | 0.474 | 0.0293 | 0.05 |
| Category Fluency | T-PREF-FD | 0 | 0 | 0 | 0 | 0 | 0 |
| Category Fluency | T-PREM-FD | 0 | 0 | 0 | 0 | 0 | 0 |
| Category Fluency | T-PREC-FD | 0 | 0 | 0 | 0 | 0 | 0 |
| Category Fluency | T-POSTC-FD | 0 | 0 | 0 | 0 | 0 | 0 |
| Category Fluency | T-PAR-FD | 0 | 0 | 0 | 0 | 0 | 0 |
| Category Fluency | T-OCC-FD | 0 | 0 | 0 | 0 | 0 | 0 |
| Category Fluency | ATR-FD | 0.23 | 0.113 | 0.004 | 0.455 | 0.046 | 0.04 |
| Category Fluency | STR-FD | 0 | 0 | 0 | 0 | 0 | 0 |
| Category Fluency | OR-FD | 0 | 0 | 0 | 0 | 0 | 0 |
| Category Fluency | FPT-FD | 0 | 0 | 0 | 0 | 0 | 0 |
| Category Fluency | CST-FD | 0 | 0 | 0 | 0 | 0 | 0 |
| Category Fluency | POPT-FD | 0 | 0 | 0 | 0 | 0 | 0 |
| Category Fluency | CC-1-FD | 0 | 0 | 0 | 0 | 0 | 0 |
| Category Fluency | CC-G-FD | 0 | 0 | 0 | 0 | 0 | 0 |
| Category Fluency | CC-3-FD | 0 | 0 | 0 | 0 | 0 | 0 |
| Category Fluency | CC-4-FD | 0 | 0 | 0 | 0 | 0 | 0 |
| Category Fluency | CC-5-FD | 0 | 0 | 0 | 0 | 0 | 0 |
| Category Fluency | CC-6-FD | 0 | 0 | 0 | 0 | 0 | 0 |
| Category Fluency | CC-7-FD | 0 | 0 | 0 | 0 | 0 | 0 |
| Category Fluency | CA-FD | 0 | 0 | 0 | 0 | 0 | 0 |
| Category Fluency | CG-FD | 0 | 0 | 0 | 0 | 0 | 0 |
| Category Fluency | AF-FC | 0 | 0 | 0 | 0 | 0 | 0 |
| Category Fluency | UF-FC | 0 | 0 | 0 | 0 | 0 | 0 |
| Category Fluency | IFO-FC | 0 | 0 | 0 | 0 | 0 | 0 |
| Category Fluency | MLF-FC | 0 | 0 | 0 | 0 | 0 | 0 |
| Category Fluency | ILF-FC | 0 | 0 | 0 | 0 | 0 | 0 |
| Category Fluency | SLF-I-FC | 0 | 0 | 0 | 0 | 0 | 0 |
| Category Fluency | SLF-II-FC | 0 | 0 | 0 | 0 | 0 | 0 |
| Category Fluency | SLF-III-FC | 0 | 0 | 0 | 0 | 0 | 0 |
| Category Fluency | T-PREF-FC | 0 | 0 | 0 | 0 | 0 | 0 |
| Category Fluency | T-PREM-FC | 0 | 0 | 0 | 0 | 0 | 0 |
| Category Fluency | T-PREC-FC | 0 | 0 | 0 | 0 | 0 | 0 |
| Category Fluency | T-POSTC-FC | 0 | 0 | 0 | 0 | 0 | 0 |
| Category Fluency | T-PAR-FC | 0 | 0 | 0 | 0 | 0 | 0 |
| Category Fluency | T-OCC-FC | 0 | 0 | 0 | 0 | 0 | 0 |
| Category Fluency | ATR-FC | 0 | 0 | 0 | 0 | 0 | 0 |
| Category Fluency | STR-FC | 0 | 0 | 0 | 0 | 0 | 0 |
| Category Fluency | OR-FC | 0 | 0 | 0 | 0 | 0 | 0 |
| Category Fluency | FPT-FC | 0 | 0 | 0 | 0 | 0 | 0 |
| Category Fluency | CST-FC | 0 | 0 | 0 | 0 | 0 | 0 |
| Category Fluency | POPT-FC | 0 | 0 | 0 | 0 | 0 | 0 |
| Category Fluency | CC-1-FC | 0 | 0 | 0 | 0 | 0 | 0 |
| Category Fluency | CC-G-FC | 0 | 0 | 0 | 0 | 0 | 0 |
| Category Fluency | CC-3-FC | 0 | 0 | 0 | 0 | 0 | 0 |
| Category Fluency | CC-4-FC | 0 | 0 | 0 | 0 | 0 | 0 |
| Category Fluency | CC-5-FC | 0 | 0 | 0 | 0 | 0 | 0 |
| Category Fluency | CC-6-FC | 0 | 0 | 0 | 0 | 0 | 0 |
| Category Fluency | CC-7-FC | 0 | 0 | 0 | 0 | 0 | 0 |
| Category Fluency | CA-FC | 0.24 | 0.113 | 0.016 | 0.465 | 0.0364 | 0.045 |
| Category Fluency | CG-FC | 0 | 0 | 0 | 0 | 0 | 0 |
| Boston Naming Test | NfL | -0.308 | 0.111 | -0.528 | -0.087 | 0.00684 | 0.082 |
| Boston Naming Test | GFAP | 0 | 0 | 0 | 0 | 0 | 0 |
| Boston Naming Test | Ptau217 | -0.268 | 0.112 | -0.492 | -0.045 | 0.019 | 0.06 |
| Boston Naming Test | AD-Cortical Thickness | 0.254 | 0.112 | 0.03 | 0.478 | 0.0267 | 0.052 |
| Boston Naming Test | MSMD | 0 | 0 | 0 | 0 | 0 | 0 |
| Boston Naming Test | WMHvol | 0 | 0 | 0 | 0 | 0 | 0 |
| Boston Naming Test | AF-FD | 0 | 0 | 0 | 0 | 0 | 0 |
| Boston Naming Test | UF-FD | 0 | 0 | 0 | 0 | 0 | 0 |
| Boston Naming Test | IFO-FD | 0 | 0 | 0 | 0 | 0 | 0 |
| Boston Naming Test | MLF-FD | 0 | 0 | 0 | 0 | 0 | 0 |
| Boston Naming Test | ILF-FD | 0 | 0 | 0 | 0 | 0 | 0 |
| Boston Naming Test | SLF-I-FD | 0 | 0 | 0 | 0 | 0 | 0 |
| Boston Naming Test | SLF-II-FD | 0 | 0 | 0 | 0 | 0 | 0 |
| Boston Naming Test | SLF-III-FD | 0 | 0 | 0 | 0 | 0 | 0 |
| Boston Naming Test | T-PREF-FD | 0 | 0 | 0 | 0 | 0 | 0 |
| Boston Naming Test | T-PREM-FD | 0 | 0 | 0 | 0 | 0 | 0 |
| Boston Naming Test | T-PREC-FD | 0 | 0 | 0 | 0 | 0 | 0 |
| Boston Naming Test | T-POSTC-FD | 0 | 0 | 0 | 0 | 0 | 0 |
| Boston Naming Test | T-PAR-FD | 0 | 0 | 0 | 0 | 0 | 0 |
| Boston Naming Test | T-OCC-FD | 0 | 0 | 0 | 0 | 0 | 0 |
| Boston Naming Test | ATR-FD | 0 | 0 | 0 | 0 | 0 | 0 |
| Boston Naming Test | STR-FD | 0 | 0 | 0 | 0 | 0 | 0 |
| Boston Naming Test | OR-FD | 0 | 0 | 0 | 0 | 0 | 0 |
| Boston Naming Test | FPT-FD | 0 | 0 | 0 | 0 | 0 | 0 |
| Boston Naming Test | CST-FD | 0 | 0 | 0 | 0 | 0 | 0 |
| Boston Naming Test | POPT-FD | 0 | 0 | 0 | 0 | 0 | 0 |
| Boston Naming Test | CC-1-FD | 0 | 0 | 0 | 0 | 0 | 0 |
| Boston Naming Test | CC-G-FD | 0 | 0 | 0 | 0 | 0 | 0 |
| Boston Naming Test | CC-3-FD | 0 | 0 | 0 | 0 | 0 | 0 |
| Boston Naming Test | CC-4-FD | 0 | 0 | 0 | 0 | 0 | 0 |
| Boston Naming Test | CC-5-FD | 0 | 0 | 0 | 0 | 0 | 0 |
| Boston Naming Test | CC-6-FD | 0 | 0 | 0 | 0 | 0 | 0 |
| Boston Naming Test | CC-7-FD | 0 | 0 | 0 | 0 | 0 | 0 |
| Boston Naming Test | CA-FD | 0 | 0 | 0 | 0 | 0 | 0 |
| Boston Naming Test | CG-FD | 0 | 0 | 0 | 0 | 0 | 0 |
| Boston Naming Test | AF-FC | 0 | 0 | 0 | 0 | 0 | 0 |
| Boston Naming Test | UF-FC | 0 | 0 | 0 | 0 | 0 | 0 |
| Boston Naming Test | IFO-FC | 0 | 0 | 0 | 0 | 0 | 0 |
| Boston Naming Test | MLF-FC | 0 | 0 | 0 | 0 | 0 | 0 |
| Boston Naming Test | ILF-FC | 0 | 0 | 0 | 0 | 0 | 0 |
| Boston Naming Test | SLF-I-FC | 0 | 0 | 0 | 0 | 0 | 0 |
| Boston Naming Test | SLF-II-FC | 0 | 0 | 0 | 0 | 0 | 0 |
| Boston Naming Test | SLF-III-FC | 0 | 0 | 0 | 0 | 0 | 0 |
| Boston Naming Test | T-PREF-FC | 0 | 0 | 0 | 0 | 0 | 0 |
| Boston Naming Test | T-PREM-FC | 0 | 0 | 0 | 0 | 0 | 0 |
| Boston Naming Test | T-PREC-FC | 0 | 0 | 0 | 0 | 0 | 0 |
| Boston Naming Test | T-POSTC-FC | 0 | 0 | 0 | 0 | 0 | 0 |
| Boston Naming Test | T-PAR-FC | 0 | 0 | 0 | 0 | 0 | 0 |
| Boston Naming Test | T-OCC-FC | 0 | 0 | 0 | 0 | 0 | 0 |
| Boston Naming Test | ATR-FC | 0 | 0 | 0 | 0 | 0 | 0 |
| Boston Naming Test | STR-FC | 0 | 0 | 0 | 0 | 0 | 0 |
| Boston Naming Test | OR-FC | 0 | 0 | 0 | 0 | 0 | 0 |
| Boston Naming Test | FPT-FC | 0 | 0 | 0 | 0 | 0 | 0 |
| Boston Naming Test | CST-FC | 0 | 0 | 0 | 0 | 0 | 0 |
| Boston Naming Test | POPT-FC | 0 | 0 | 0 | 0 | 0 | 0 |
| Boston Naming Test | CC-1-FC | 0 | 0 | 0 | 0 | 0 | 0 |
| Boston Naming Test | CC-G-FC | 0 | 0 | 0 | 0 | 0 | 0 |
| Boston Naming Test | CC-3-FC | 0 | 0 | 0 | 0 | 0 | 0 |
| Boston Naming Test | CC-4-FC | 0 | 0 | 0 | 0 | 0 | 0 |
| Boston Naming Test | CC-5-FC | 0 | 0 | 0 | 0 | 0 | 0 |
| Boston Naming Test | CC-6-FC | 0 | 0 | 0 | 0 | 0 | 0 |
| Boston Naming Test | CC-7-FC | 0 | 0 | 0 | 0 | 0 | 0 |
| Boston Naming Test | CA-FC | 0.231 | 0.113 | 0.005 | 0.456 | 0.0449 | 0.04 |
| Boston Naming Test | CG-FC | 0 | 0 | 0 | 0 | 0 | 0 |
| MMSE | NfL | 0 | 0 | 0 | 0 | 0 | 0 |
| MMSE | GFAP | 0 | 0 | 0 | 0 | 0 | 0 |
| MMSE | Ptau217 | -0.386 | 0.107 | -0.6 | -0.171 | <0.001 | 0.138 |
| MMSE | AD-Cortical Thickness | 0.263 | 0.114 | 0.036 | 0.491 | 0.0241 | 0.055 |
| MMSE | MSMD | 0 | 0 | 0 | 0 | 0 | 0 |
| MMSE | WMHvol | 0 | 0 | 0 | 0 | 0 | 0 |
| MMSE | AF-FD | 0 | 0 | 0 | 0 | 0 | 0 |
| MMSE | UF-FD | 0 | 0 | 0 | 0 | 0 | 0 |
| MMSE | IFO-FD | 0 | 0 | 0 | 0 | 0 | 0 |
| MMSE | MLF-FD | 0 | 0 | 0 | 0 | 0 | 0 |
| MMSE | ILF-FD | 0 | 0 | 0 | 0 | 0 | 0 |
| MMSE | SLF-I-FD | 0 | 0 | 0 | 0 | 0 | 0 |
| MMSE | SLF-II-FD | 0 | 0 | 0 | 0 | 0 | 0 |
| MMSE | SLF-III-FD | 0 | 0 | 0 | 0 | 0 | 0 |
| MMSE | T-PREF-FD | 0 | 0 | 0 | 0 | 0 | 0 |
| MMSE | T-PREM-FD | 0 | 0 | 0 | 0 | 0 | 0 |
| MMSE | T-PREC-FD | 0 | 0 | 0 | 0 | 0 | 0 |
| MMSE | T-POSTC-FD | 0 | 0 | 0 | 0 | 0 | 0 |
| MMSE | T-PAR-FD | 0 | 0 | 0 | 0 | 0 | 0 |
| MMSE | T-OCC-FD | 0 | 0 | 0 | 0 | 0 | 0 |
| MMSE | ATR-FD | 0 | 0 | 0 | 0 | 0 | 0 |
| MMSE | STR-FD | 0 | 0 | 0 | 0 | 0 | 0 |
| MMSE | OR-FD | 0 | 0 | 0 | 0 | 0 | 0 |
| MMSE | FPT-FD | 0 | 0 | 0 | 0 | 0 | 0 |
| MMSE | CST-FD | 0 | 0 | 0 | 0 | 0 | 0 |
| MMSE | POPT-FD | 0 | 0 | 0 | 0 | 0 | 0 |
| MMSE | CC-1-FD | 0 | 0 | 0 | 0 | 0 | 0 |
| MMSE | CC-G-FD | 0 | 0 | 0 | 0 | 0 | 0 |
| MMSE | CC-3-FD | 0 | 0 | 0 | 0 | 0 | 0 |
| MMSE | CC-4-FD | 0 | 0 | 0 | 0 | 0 | 0 |
| MMSE | CC-5-FD | 0 | 0 | 0 | 0 | 0 | 0 |
| MMSE | CC-6-FD | 0 | 0 | 0 | 0 | 0 | 0 |
| MMSE | CC-7-FD | 0 | 0 | 0 | 0 | 0 | 0 |
| MMSE | CA-FD | 0 | 0 | 0 | 0 | 0 | 0 |
| MMSE | CG-FD | 0 | 0 | 0 | 0 | 0 | 0 |
| MMSE | AF-FC | 0 | 0 | 0 | 0 | 0 | 0 |
| MMSE | UF-FC | 0 | 0 | 0 | 0 | 0 | 0 |
| MMSE | IFO-FC | 0 | 0 | 0 | 0 | 0 | 0 |
| MMSE | MLF-FC | 0 | 0 | 0 | 0 | 0 | 0 |
| MMSE | ILF-FC | 0 | 0 | 0 | 0 | 0 | 0 |
| MMSE | SLF-I-FC | 0 | 0 | 0 | 0 | 0 | 0 |
| MMSE | SLF-II-FC | 0 | 0 | 0 | 0 | 0 | 0 |
| MMSE | SLF-III-FC | 0 | 0 | 0 | 0 | 0 | 0 |
| MMSE | T-PREF-FC | 0 | 0 | 0 | 0 | 0 | 0 |
| MMSE | T-PREM-FC | 0 | 0 | 0 | 0 | 0 | 0 |
| MMSE | T-PREC-FC | 0 | 0 | 0 | 0 | 0 | 0 |
| MMSE | T-POSTC-FC | 0 | 0 | 0 | 0 | 0 | 0 |
| MMSE | T-PAR-FC | 0 | 0 | 0 | 0 | 0 | 0 |
| MMSE | T-OCC-FC | 0 | 0 | 0 | 0 | 0 | 0 |
| MMSE | ATR-FC | 0 | 0 | 0 | 0 | 0 | 0 |
| MMSE | STR-FC | 0 | 0 | 0 | 0 | 0 | 0 |
| MMSE | OR-FC | 0 | 0 | 0 | 0 | 0 | 0 |
| MMSE | FPT-FC | 0 | 0 | 0 | 0 | 0 | 0 |
| MMSE | CST-FC | 0 | 0 | 0 | 0 | 0 | 0 |
| MMSE | POPT-FC | 0 | 0 | 0 | 0 | 0 | 0 |
| MMSE | CC-1-FC | 0 | 0 | 0 | 0 | 0 | 0 |
| MMSE | CC-G-FC | 0 | 0 | 0 | 0 | 0 | 0 |
| MMSE | CC-3-FC | 0 | 0 | 0 | 0 | 0 | 0 |
| MMSE | CC-4-FC | 0 | 0 | 0 | 0 | 0 | 0 |
| MMSE | CC-5-FC | 0 | 0 | 0 | 0 | 0 | 0 |
| MMSE | CC-6-FC | 0 | 0 | 0 | 0 | 0 | 0 |
| MMSE | CC-7-FC | 0 | 0 | 0 | 0 | 0 | 0 |
| MMSE | CA-FC | 0 | 0 | 0 | 0 | 0 | 0 |
| MMSE | CG-FC | 0 | 0 | 0 | 0 | 0 | 0 |
| Word List Learning | NfL | -0.266 | 0.112 | -0.49 | -0.043 | 0.02 | 0.058 |
| Word List Learning | GFAP | 0 | 0 | 0 | 0 | 0 | 0 |
| Word List Learning | Ptau217 | -0.377 | 0.108 | -0.591 | -0.162 | <0.001 | 0.13 |
| Word List Learning | AD-Cortical Thickness | 0.443 | 0.104 | 0.236 | 0.651 | <0.001 | 0.186 |
| Word List Learning | MSMD | 0 | 0 | 0 | 0 | 0 | 0 |
| Word List Learning | WMHvol | 0 | 0 | 0 | 0 | 0 | 0 |
| Word List Learning | AF-FD | 0 | 0 | 0 | 0 | 0 | 0 |
| Word List Learning | UF-FD | 0 | 0 | 0 | 0 | 0 | 0 |
| Word List Learning | IFO-FD | 0 | 0 | 0 | 0 | 0 | 0 |
| Word List Learning | MLF-FD | 0 | 0 | 0 | 0 | 0 | 0 |
| Word List Learning | ILF-FD | 0 | 0 | 0 | 0 | 0 | 0 |
| Word List Learning | SLF-I-FD | 0 | 0 | 0 | 0 | 0 | 0 |
| Word List Learning | SLF-II-FD | 0 | 0 | 0 | 0 | 0 | 0 |
| Word List Learning | SLF-III-FD | 0 | 0 | 0 | 0 | 0 | 0 |
| Word List Learning | T-PREF-FD | 0 | 0 | 0 | 0 | 0 | 0 |
| Word List Learning | T-PREM-FD | 0 | 0 | 0 | 0 | 0 | 0 |
| Word List Learning | T-PREC-FD | 0 | 0 | 0 | 0 | 0 | 0 |
| Word List Learning | T-POSTC-FD | 0 | 0 | 0 | 0 | 0 | 0 |
| Word List Learning | T-PAR-FD | 0 | 0 | 0 | 0 | 0 | 0 |
| Word List Learning | T-OCC-FD | 0 | 0 | 0 | 0 | 0 | 0 |
| Word List Learning | ATR-FD | 0 | 0 | 0 | 0 | 0 | 0 |
| Word List Learning | STR-FD | 0 | 0 | 0 | 0 | 0 | 0 |
| Word List Learning | OR-FD | 0 | 0 | 0 | 0 | 0 | 0 |
| Word List Learning | FPT-FD | 0 | 0 | 0 | 0 | 0 | 0 |
| Word List Learning | CST-FD | 0 | 0 | 0 | 0 | 0 | 0 |
| Word List Learning | POPT-FD | 0 | 0 | 0 | 0 | 0 | 0 |
| Word List Learning | CC-1-FD | 0 | 0 | 0 | 0 | 0 | 0 |
| Word List Learning | CC-G-FD | 0 | 0 | 0 | 0 | 0 | 0 |
| Word List Learning | CC-3-FD | 0 | 0 | 0 | 0 | 0 | 0 |
| Word List Learning | CC-4-FD | 0 | 0 | 0 | 0 | 0 | 0 |
| Word List Learning | CC-5-FD | 0 | 0 | 0 | 0 | 0 | 0 |
| Word List Learning | CC-6-FD | 0 | 0 | 0 | 0 | 0 | 0 |
| Word List Learning | CC-7-FD | 0 | 0 | 0 | 0 | 0 | 0 |
| Word List Learning | CA-FD | 0 | 0 | 0 | 0 | 0 | 0 |
| Word List Learning | CG-FD | 0 | 0 | 0 | 0 | 0 | 0 |
| Word List Learning | AF-FC | 0.262 | 0.112 | 0.039 | 0.486 | 0.0221 | 0.056 |
| Word List Learning | UF-FC | 0.372 | 0.108 | 0.157 | 0.587 | <0.001 | 0.127 |
| Word List Learning | IFO-FC | 0 | 0 | 0 | 0 | 0 | 0 |
| Word List Learning | MLF-FC | 0 | 0 | 0 | 0 | 0 | 0 |
| Word List Learning | ILF-FC | 0 | 0 | 0 | 0 | 0 | 0 |
| Word List Learning | SLF-I-FC | 0.24 | 0.113 | 0.016 | 0.465 | 0.0365 | 0.045 |
| Word List Learning | SLF-II-FC | 0.302 | 0.111 | 0.081 | 0.523 | 0.00808 | 0.079 |
| Word List Learning | SLF-III-FC | 0 | 0 | 0 | 0 | 0 | 0 |
| Word List Learning | T-PREF-FC | 0.27 | 0.112 | 0.047 | 0.493 | 0.0181 | 0.061 |
| Word List Learning | T-PREM-FC | 0.238 | 0.113 | 0.013 | 0.463 | 0.0387 | 0.044 |
| Word List Learning | T-PREC-FC | 0 | 0 | 0 | 0 | 0 | 0 |
| Word List Learning | T-POSTC-FC | 0 | 0 | 0 | 0 | 0 | 0 |
| Word List Learning | T-PAR-FC | 0 | 0 | 0 | 0 | 0 | 0 |
| Word List Learning | T-OCC-FC | 0 | 0 | 0 | 0 | 0 | 0 |
| Word List Learning | ATR-FC | 0.229 | 0.113 | 0.004 | 0.455 | 0.0465 | 0.04 |
| Word List Learning | STR-FC | 0 | 0 | 0 | 0 | 0 | 0 |
| Word List Learning | OR-FC | 0 | 0 | 0 | 0 | 0 | 0 |
| Word List Learning | FPT-FC | 0.251 | 0.113 | 0.027 | 0.476 | 0.0284 | 0.051 |
| Word List Learning | CST-FC | 0 | 0 | 0 | 0 | 0 | 0 |
| Word List Learning | POPT-FC | 0 | 0 | 0 | 0 | 0 | 0 |
| Word List Learning | CC-1-FC | 0.262 | 0.112 | 0.039 | 0.486 | 0.022 | 0.056 |
| Word List Learning | CC-G-FC | 0.265 | 0.112 | 0.042 | 0.489 | 0.0205 | 0.058 |
| Word List Learning | CC-3-FC | 0.245 | 0.113 | 0.02 | 0.469 | 0.033 | 0.047 |
| Word List Learning | CC-4-FC | 0 | 0 | 0 | 0 | 0 | 0 |
| Word List Learning | CC-5-FC | 0 | 0 | 0 | 0 | 0 | 0 |
| Word List Learning | CC-6-FC | 0 | 0 | 0 | 0 | 0 | 0 |
| Word List Learning | CC-7-FC | 0 | 0 | 0 | 0 | 0 | 0 |
| Word List Learning | CA-FC | 0.391 | 0.107 | 0.178 | 0.604 | <0.001 | 0.141 |
| Word List Learning | CG-FC | 0.279 | 0.112 | 0.057 | 0.501 | 0.0147 | 0.065 |
| Letter Fluency | NfL | 0 | 0 | 0 | 0 | 0 | 0 |
| Letter Fluency | GFAP | 0 | 0 | 0 | 0 | 0 | 0 |
| Letter Fluency | Ptau217 | 0 | 0 | 0 | 0 | 0 | 0 |
| Letter Fluency | AD-Cortical Thickness | 0 | 0 | 0 | 0 | 0 | 0 |
| Letter Fluency | MSMD | 0 | 0 | 0 | 0 | 0 | 0 |
| Letter Fluency | WMHvol | 0 | 0 | 0 | 0 | 0 | 0 |
| Letter Fluency | AF-FD | 0 | 0 | 0 | 0 | 0 | 0 |
| Letter Fluency | UF-FD | 0 | 0 | 0 | 0 | 0 | 0 |
| Letter Fluency | IFO-FD | 0 | 0 | 0 | 0 | 0 | 0 |
| Letter Fluency | MLF-FD | 0 | 0 | 0 | 0 | 0 | 0 |
| Letter Fluency | ILF-FD | 0 | 0 | 0 | 0 | 0 | 0 |
| Letter Fluency | SLF-I-FD | 0 | 0 | 0 | 0 | 0 | 0 |
| Letter Fluency | SLF-II-FD | 0 | 0 | 0 | 0 | 0 | 0 |
| Letter Fluency | SLF-III-FD | 0 | 0 | 0 | 0 | 0 | 0 |
| Letter Fluency | T-PREF-FD | 0 | 0 | 0 | 0 | 0 | 0 |
| Letter Fluency | T-PREM-FD | 0 | 0 | 0 | 0 | 0 | 0 |
| Letter Fluency | T-PREC-FD | 0 | 0 | 0 | 0 | 0 | 0 |
| Letter Fluency | T-POSTC-FD | 0 | 0 | 0 | 0 | 0 | 0 |
| Letter Fluency | T-PAR-FD | 0 | 0 | 0 | 0 | 0 | 0 |
| Letter Fluency | T-OCC-FD | 0 | 0 | 0 | 0 | 0 | 0 |
| Letter Fluency | ATR-FD | 0 | 0 | 0 | 0 | 0 | 0 |
| Letter Fluency | STR-FD | 0 | 0 | 0 | 0 | 0 | 0 |
| Letter Fluency | OR-FD | 0 | 0 | 0 | 0 | 0 | 0 |
| Letter Fluency | FPT-FD | 0 | 0 | 0 | 0 | 0 | 0 |
| Letter Fluency | CST-FD | 0 | 0 | 0 | 0 | 0 | 0 |
| Letter Fluency | POPT-FD | 0 | 0 | 0 | 0 | 0 | 0 |
| Letter Fluency | CC-1-FD | 0 | 0 | 0 | 0 | 0 | 0 |
| Letter Fluency | CC-G-FD | 0 | 0 | 0 | 0 | 0 | 0 |
| Letter Fluency | CC-3-FD | 0 | 0 | 0 | 0 | 0 | 0 |
| Letter Fluency | CC-4-FD | 0 | 0 | 0 | 0 | 0 | 0 |
| Letter Fluency | CC-5-FD | 0 | 0 | 0 | 0 | 0 | 0 |
| Letter Fluency | CC-6-FD | 0 | 0 | 0 | 0 | 0 | 0 |
| Letter Fluency | CC-7-FD | 0 | 0 | 0 | 0 | 0 | 0 |
| Letter Fluency | CA-FD | 0 | 0 | 0 | 0 | 0 | 0 |
| Letter Fluency | CG-FD | 0 | 0 | 0 | 0 | 0 | 0 |
| Letter Fluency | AF-FC | 0 | 0 | 0 | 0 | 0 | 0 |
| Letter Fluency | UF-FC | 0 | 0 | 0 | 0 | 0 | 0 |
| Letter Fluency | IFO-FC | 0 | 0 | 0 | 0 | 0 | 0 |
| Letter Fluency | MLF-FC | 0 | 0 | 0 | 0 | 0 | 0 |
| Letter Fluency | ILF-FC | 0 | 0 | 0 | 0 | 0 | 0 |
| Letter Fluency | SLF-I-FC | 0 | 0 | 0 | 0 | 0 | 0 |
| Letter Fluency | SLF-II-FC | 0 | 0 | 0 | 0 | 0 | 0 |
| Letter Fluency | SLF-III-FC | 0 | 0 | 0 | 0 | 0 | 0 |
| Letter Fluency | T-PREF-FC | 0 | 0 | 0 | 0 | 0 | 0 |
| Letter Fluency | T-PREM-FC | 0 | 0 | 0 | 0 | 0 | 0 |
| Letter Fluency | T-PREC-FC | 0 | 0 | 0 | 0 | 0 | 0 |
| Letter Fluency | T-POSTC-FC | 0 | 0 | 0 | 0 | 0 | 0 |
| Letter Fluency | T-PAR-FC | 0 | 0 | 0 | 0 | 0 | 0 |
| Letter Fluency | T-OCC-FC | 0 | 0 | 0 | 0 | 0 | 0 |
| Letter Fluency | ATR-FC | 0 | 0 | 0 | 0 | 0 | 0 |
| Letter Fluency | STR-FC | 0 | 0 | 0 | 0 | 0 | 0 |
| Letter Fluency | OR-FC | 0 | 0 | 0 | 0 | 0 | 0 |
| Letter Fluency | FPT-FC | 0 | 0 | 0 | 0 | 0 | 0 |
| Letter Fluency | CST-FC | 0 | 0 | 0 | 0 | 0 | 0 |
| Letter Fluency | POPT-FC | 0 | 0 | 0 | 0 | 0 | 0 |
| Letter Fluency | CC-1-FC | 0 | 0 | 0 | 0 | 0 | 0 |
| Letter Fluency | CC-G-FC | 0 | 0 | 0 | 0 | 0 | 0 |
| Letter Fluency | CC-3-FC | 0 | 0 | 0 | 0 | 0 | 0 |
| Letter Fluency | CC-4-FC | 0 | 0 | 0 | 0 | 0 | 0 |
| Letter Fluency | CC-5-FC | 0 | 0 | 0 | 0 | 0 | 0 |
| Letter Fluency | CC-6-FC | 0 | 0 | 0 | 0 | 0 | 0 |
| Letter Fluency | CC-7-FC | 0 | 0 | 0 | 0 | 0 | 0 |
| Letter Fluency | CA-FC | 0 | 0 | 0 | 0 | 0 | 0 |
| Letter Fluency | CG-FC | 0 | 0 | 0 | 0 | 0 | 0 |
| TMTA | NfL | -0.338 | 0.109 | -0.556 | -0.12 | 0.00281 | 0.102 |
| TMTA | GFAP | -0.305 | 0.111 | -0.526 | -0.084 | 0.00739 | 0.081 |
| TMTA | Ptau217 | 0 | 0 | 0 | 0 | 0 | 0 |
| TMTA | AD-Cortical Thickness | 0 | 0 | 0 | 0 | 0 | 0 |
| TMTA | MSMD | -0.277 | 0.112 | -0.499 | -0.054 | 0.0155 | 0.064 |
| TMTA | WMHvol | 0 | 0 | 0 | 0 | 0 | 0 |
| TMTA | AF-FD | 0 | 0 | 0 | 0 | 0 | 0 |
| TMTA | UF-FD | 0 | 0 | 0 | 0 | 0 | 0 |
| TMTA | IFO-FD | 0 | 0 | 0 | 0 | 0 | 0 |
| TMTA | MLF-FD | 0 | 0 | 0 | 0 | 0 | 0 |
| TMTA | ILF-FD | 0 | 0 | 0 | 0 | 0 | 0 |
| TMTA | SLF-I-FD | 0 | 0 | 0 | 0 | 0 | 0 |
| TMTA | SLF-II-FD | 0 | 0 | 0 | 0 | 0 | 0 |
| TMTA | SLF-III-FD | 0.235 | 0.113 | 0.01 | 0.46 | 0.0413 | 0.042 |
| TMTA | T-PREF-FD | 0 | 0 | 0 | 0 | 0 | 0 |
| TMTA | T-PREM-FD | 0 | 0 | 0 | 0 | 0 | 0 |
| TMTA | T-PREC-FD | 0 | 0 | 0 | 0 | 0 | 0 |
| TMTA | T-POSTC-FD | 0 | 0 | 0 | 0 | 0 | 0 |
| TMTA | T-PAR-FD | 0 | 0 | 0 | 0 | 0 | 0 |
| TMTA | T-OCC-FD | 0 | 0 | 0 | 0 | 0 | 0 |
| TMTA | ATR-FD | 0.25 | 0.113 | 0.026 | 0.474 | 0.0295 | 0.05 |
| TMTA | STR-FD | 0 | 0 | 0 | 0 | 0 | 0 |
| TMTA | OR-FD | 0 | 0 | 0 | 0 | 0 | 0 |
| TMTA | FPT-FD | 0 | 0 | 0 | 0 | 0 | 0 |
| TMTA | CST-FD | 0 | 0 | 0 | 0 | 0 | 0 |
| TMTA | POPT-FD | 0 | 0 | 0 | 0 | 0 | 0 |
| TMTA | CC-1-FD | 0 | 0 | 0 | 0 | 0 | 0 |
| TMTA | CC-G-FD | 0 | 0 | 0 | 0 | 0 | 0 |
| TMTA | CC-3-FD | 0.236 | 0.113 | 0.011 | 0.461 | 0.0403 | 0.043 |
| TMTA | CC-4-FD | 0 | 0 | 0 | 0 | 0 | 0 |
| TMTA | CC-5-FD | 0 | 0 | 0 | 0 | 0 | 0 |
| TMTA | CC-6-FD | 0 | 0 | 0 | 0 | 0 | 0 |
| TMTA | CC-7-FD | 0 | 0 | 0 | 0 | 0 | 0 |
| TMTA | CA-FD | 0 | 0 | 0 | 0 | 0 | 0 |
| TMTA | CG-FD | 0.27 | 0.112 | 0.047 | 0.493 | 0.0183 | 0.06 |
| TMTA | AF-FC | 0 | 0 | 0 | 0 | 0 | 0 |
| TMTA | UF-FC | 0 | 0 | 0 | 0 | 0 | 0 |
| TMTA | IFO-FC | 0 | 0 | 0 | 0 | 0 | 0 |
| TMTA | MLF-FC | 0 | 0 | 0 | 0 | 0 | 0 |
| TMTA | ILF-FC | 0 | 0 | 0 | 0 | 0 | 0 |
| TMTA | SLF-I-FC | 0 | 0 | 0 | 0 | 0 | 0 |
| TMTA | SLF-II-FC | 0 | 0 | 0 | 0 | 0 | 0 |
| TMTA | SLF-III-FC | 0 | 0 | 0 | 0 | 0 | 0 |
| TMTA | T-PREF-FC | 0 | 0 | 0 | 0 | 0 | 0 |
| TMTA | T-PREM-FC | 0 | 0 | 0 | 0 | 0 | 0 |
| TMTA | T-PREC-FC | 0 | 0 | 0 | 0 | 0 | 0 |
| TMTA | T-POSTC-FC | 0 | 0 | 0 | 0 | 0 | 0 |
| TMTA | T-PAR-FC | 0 | 0 | 0 | 0 | 0 | 0 |
| TMTA | T-OCC-FC | 0 | 0 | 0 | 0 | 0 | 0 |
| TMTA | ATR-FC | 0 | 0 | 0 | 0 | 0 | 0 |
| TMTA | STR-FC | 0 | 0 | 0 | 0 | 0 | 0 |
| TMTA | OR-FC | 0 | 0 | 0 | 0 | 0 | 0 |
| TMTA | FPT-FC | 0 | 0 | 0 | 0 | 0 | 0 |
| TMTA | CST-FC | 0 | 0 | 0 | 0 | 0 | 0 |
| TMTA | POPT-FC | 0 | 0 | 0 | 0 | 0 | 0 |
| TMTA | CC-1-FC | 0 | 0 | 0 | 0 | 0 | 0 |
| TMTA | CC-G-FC | 0 | 0 | 0 | 0 | 0 | 0 |
| TMTA | CC-3-FC | 0 | 0 | 0 | 0 | 0 | 0 |
| TMTA | CC-4-FC | 0 | 0 | 0 | 0 | 0 | 0 |
| TMTA | CC-5-FC | 0 | 0 | 0 | 0 | 0 | 0 |
| TMTA | CC-6-FC | 0 | 0 | 0 | 0 | 0 | 0 |
| TMTA | CC-7-FC | 0 | 0 | 0 | 0 | 0 | 0 |
| TMTA | CA-FC | 0.26 | 0.112 | 0.036 | 0.484 | 0.0234 | 0.055 |
| TMTA | CG-FC | 0 | 0 | 0 | 0 | 0 | 0 |
| TMTB | NfL | -0.373 | 0.109 | -0.591 | -0.156 | 0.00104 | 0.131 |
| TMTB | GFAP | -0.32 | 0.125 | -0.569 | -0.071 | 0.0125 | 0.073 |
| TMTB | Ptau217 | 0 | 0 | 0 | 0 | 0 | 0 |
| TMTB | AD-Cortical Thickness | 0 | 0 | 0 | 0 | 0 | 0 |
| TMTB | MSMD | -0.353 | 0.11 | -0.573 | -0.133 | 0.00207 | 0.115 |
| TMTB | WMHvol | 0 | 0 | 0 | 0 | 0 | 0 |
| TMTB | AF-FD | 0.357 | 0.113 | 0.132 | 0.582 | 0.00232 | 0.112 |
| TMTB | UF-FD | 0 | 0 | 0 | 0 | 0 | 0 |
| TMTB | IFO-FD | 0.299 | 0.116 | 0.068 | 0.531 | 0.0121 | 0.074 |
| TMTB | MLF-FD | 0 | 0 | 0 | 0 | 0 | 0 |
| TMTB | ILF-FD | 0 | 0 | 0 | 0 | 0 | 0 |
| TMTB | SLF-I-FD | 0.385 | 0.11 | 0.165 | 0.605 | <0.001 | 0.136 |
| TMTB | SLF-II-FD | 0.307 | 0.112 | 0.083 | 0.531 | 0.00781 | 0.084 |
| TMTB | SLF-III-FD | 0.348 | 0.113 | 0.122 | 0.574 | 0.00307 | 0.106 |
| TMTB | T-PREF-FD | 0 | 0 | 0 | 0 | 0 | 0 |
| TMTB | T-PREM-FD | 0.263 | 0.116 | 0.032 | 0.494 | 0.0261 | 0.055 |
| TMTB | T-PREC-FD | 0 | 0 | 0 | 0 | 0 | 0 |
| TMTB | T-POSTC-FD | 0 | 0 | 0 | 0 | 0 | 0 |
| TMTB | T-PAR-FD | 0 | 0 | 0 | 0 | 0 | 0 |
| TMTB | T-OCC-FD | 0.282 | 0.117 | 0.048 | 0.516 | 0.0189 | 0.063 |
| TMTB | ATR-FD | 0.274 | 0.116 | 0.042 | 0.506 | 0.0214 | 0.06 |
| TMTB | STR-FD | 0 | 0 | 0 | 0 | 0 | 0 |
| TMTB | OR-FD | 0.288 | 0.117 | 0.053 | 0.522 | 0.0168 | 0.066 |
| TMTB | FPT-FD | 0 | 0 | 0 | 0 | 0 | 0 |
| TMTB | CST-FD | 0 | 0 | 0 | 0 | 0 | 0 |
| TMTB | POPT-FD | 0 | 0 | 0 | 0 | 0 | 0 |
| TMTB | CC-1-FD | 0.351 | 0.116 | 0.12 | 0.582 | 0.0034 | 0.103 |
| TMTB | CC-G-FD | 0.36 | 0.115 | 0.13 | 0.59 | 0.00264 | 0.109 |
| TMTB | CC-3-FD | 0.361 | 0.115 | 0.131 | 0.59 | 0.00257 | 0.11 |
| TMTB | CC-4-FD | 0.304 | 0.116 | 0.073 | 0.536 | 0.0107 | 0.076 |
| TMTB | CC-5-FD | 0.265 | 0.117 | 0.032 | 0.497 | 0.0264 | 0.055 |
| TMTB | CC-6-FD | 0 | 0 | 0 | 0 | 0 | 0 |
| TMTB | CC-7-FD | 0.346 | 0.115 | 0.117 | 0.575 | 0.00355 | 0.102 |
| TMTB | CA-FD | 0.303 | 0.113 | 0.077 | 0.529 | 0.00936 | 0.08 |
| TMTB | CG-FD | 0.327 | 0.116 | 0.095 | 0.558 | 0.00631 | 0.089 |
| TMTB | AF-FC | 0 | 0 | 0 | 0 | 0 | 0 |
| TMTB | UF-FC | 0.25 | 0.116 | 0.018 | 0.482 | 0.0347 | 0.049 |
| TMTB | IFO-FC | 0 | 0 | 0 | 0 | 0 | 0 |
| TMTB | MLF-FC | 0 | 0 | 0 | 0 | 0 | 0 |
| TMTB | ILF-FC | 0 | 0 | 0 | 0 | 0 | 0 |
| TMTB | SLF-I-FC | 0 | 0 | 0 | 0 | 0 | 0 |
| TMTB | SLF-II-FC | 0 | 0 | 0 | 0 | 0 | 0 |
| TMTB | SLF-III-FC | 0 | 0 | 0 | 0 | 0 | 0 |
| TMTB | T-PREF-FC | 0 | 0 | 0 | 0 | 0 | 0 |
| TMTB | T-PREM-FC | 0 | 0 | 0 | 0 | 0 | 0 |
| TMTB | T-PREC-FC | 0 | 0 | 0 | 0 | 0 | 0 |
| TMTB | T-POSTC-FC | 0 | 0 | 0 | 0 | 0 | 0 |
| TMTB | T-PAR-FC | 0 | 0 | 0 | 0 | 0 | 0 |
| TMTB | T-OCC-FC | 0 | 0 | 0 | 0 | 0 | 0 |
| TMTB | ATR-FC | 0 | 0 | 0 | 0 | 0 | 0 |
| TMTB | STR-FC | 0 | 0 | 0 | 0 | 0 | 0 |
| TMTB | OR-FC | 0 | 0 | 0 | 0 | 0 | 0 |
| TMTB | FPT-FC | 0 | 0 | 0 | 0 | 0 | 0 |
| TMTB | CST-FC | 0 | 0 | 0 | 0 | 0 | 0 |
| TMTB | POPT-FC | 0 | 0 | 0 | 0 | 0 | 0 |
| TMTB | CC-1-FC | 0 | 0 | 0 | 0 | 0 | 0 |
| TMTB | CC-G-FC | 0 | 0 | 0 | 0 | 0 | 0 |
| TMTB | CC-3-FC | 0 | 0 | 0 | 0 | 0 | 0 |
| TMTB | CC-4-FC | 0 | 0 | 0 | 0 | 0 | 0 |
| TMTB | CC-5-FC | 0 | 0 | 0 | 0 | 0 | 0 |
| TMTB | CC-6-FC | 0 | 0 | 0 | 0 | 0 | 0 |
| TMTB | CC-7-FC | 0 | 0 | 0 | 0 | 0 | 0 |
| TMTB | CA-FC | 0.295 | 0.115 | 0.066 | 0.524 | 0.0124 | 0.073 |
| TMTB | CG-FC | 0 | 0 | 0 | 0 | 0 | 0 |

**Table e-2B**: Simple linear regression models predicting neuropsychological performance (adjusted R^2^, *p* < 0.05) in ADNI.

| **Cognition** | **Predictor** | **Std. β** | **SE** | **CI lower** | **CI upper** | ***p*** | **Adj. R^2^** |
| --- | --- | --- | --- | --- | --- | --- | --- |
| ADNI-LAN | NfL | -0.433 | 0.144 | -0.725 | -0.141 | 0.00469 | 0.167 |
| ADNI-LAN | GFAP | -0.362 | 0.149 | -0.664 | -0.06 | 0.02 | 0.109 |
| ADNI-LAN | Ptau_217_ | -0.521 | 0.137 | -0.797 | -0.244 | <0.001 | 0.253 |
| ADNI-LAN | amyloid-PET | -0.497 | 0.139 | -0.778 | -0.216 | <0.001 | 0.228 |
| ADNI-LAN | tau-PET | - | - | - | - | - | - |
| ADNI-LAN | AD-Cortical Thickness | 0.339 | 0.151 | 0.034 | 0.643 | 0.0304 | 0.092 |
| ADNI-LAN | MSMD | - | - | - | - | - | - |
| ADNI-LAN | WMHvol | -0.311 | 0.152 | -0.619 | -0.003 | 0.0477 | 0.074 |
| ADNI-LAN | AF-FD | - | - | - | - | - | - |
| ADNI-LAN | UF-FD | - | - | - | - | - | - |
| ADNI-LAN | IFO-FD | - | - | - | - | - | - |
| ADNI-LAN | MLF-FD | - | - | - | - | - | - |
| ADNI-LAN | ILF-FD | - | - | - | - | - | - |
| ADNI-LAN | SLF-I-FD | - | - | - | - | - | - |
| ADNI-LAN | SLF-II-FD | - | - | - | - | - | - |
| ADNI-LAN | SLF-III-FD | - | - | - | - | - | - |
| ADNI-LAN | T-PREF-FD | - | - | - | - | - | - |
| ADNI-LAN | T-PREM-FD | - | - | - | - | - | - |
| ADNI-LAN | T-PREC-FD | - | - | - | - | - | - |
| ADNI-LAN | T-POSTC-FD | - | - | - | - | - | - |
| ADNI-LAN | T-PAR-FD | - | - | - | - | - | - |
| ADNI-LAN | T-OCC-FD | - | - | - | - | - | - |
| ADNI-LAN | ATR-FD | - | - | - | - | - | - |
| ADNI-LAN | STR-FD | - | - | - | - | - | - |
| ADNI-LAN | OR-FD | - | - | - | - | - | - |
| ADNI-LAN | FPT-FD | - | - | - | - | - | - |
| ADNI-LAN | CST-FD | - | - | - | - | - | - |
| ADNI-LAN | POPT-FD | - | - | - | - | - | - |
| ADNI-LAN | CC-1-FD | - | - | - | - | - | - |
| ADNI-LAN | CC-G-FD | - | - | - | - | - | - |
| ADNI-LAN | CC-3-FD | 0.32 | 0.152 | 0.013 | 0.627 | 0.0413 | 0.079 |
| ADNI-LAN | CC-4-FD | - | - | - | - | - | - |
| ADNI-LAN | CC-5-FD | - | - | - | - | - | - |
| ADNI-LAN | CC-6-FD | - | - | - | - | - | - |
| ADNI-LAN | CC-7-FD | - | - | - | - | - | - |
| ADNI-LAN | CA-FD | - | - | - | - | - | - |
| ADNI-LAN | CG-FD | - | - | - | - | - | - |
| ADNI-LAN | AF-FC | - | - | - | - | - | - |
| ADNI-LAN | UF-FC | - | - | - | - | - | - |
| ADNI-LAN | IFO-FC | - | - | - | - | - | - |
| ADNI-LAN | MLF-FC | - | - | - | - | - | - |
| ADNI-LAN | ILF-FC | - | - | - | - | - | - |
| ADNI-LAN | SLF-I-FC | - | - | - | - | - | - |
| ADNI-LAN | SLF-II-FC | - | - | - | - | - | - |
| ADNI-LAN | SLF-III-FC | - | - | - | - | - | - |
| ADNI-LAN | T-PREF-FC | - | - | - | - | - | - |
| ADNI-LAN | T-PREM-FC | - | - | - | - | - | - |
| ADNI-LAN | T-PREC-FC | 0.339 | 0.151 | 0.034 | 0.643 | 0.0303 | 0.092 |
| ADNI-LAN | T-POSTC-FC | - | - | - | - | - | - |
| ADNI-LAN | T-PAR-FC | - | - | - | - | - | - |
| ADNI-LAN | T-OCC-FC | - | - | - | - | - | - |
| ADNI-LAN | ATR-FC | - | - | - | - | - | - |
| ADNI-LAN | STR-FC | 0.319 | 0.152 | 0.013 | 0.626 | 0.0418 | 0.079 |
| ADNI-LAN | OR-FC | - | - | - | - | - | - |
| ADNI-LAN | FPT-FC | - | - | - | - | - | - |
| ADNI-LAN | CST-FC | - | - | - | - | - | - |
| ADNI-LAN | POPT-FC | - | - | - | - | - | - |
| ADNI-LAN | CC-1-FC | - | - | - | - | - | - |
| ADNI-LAN | CC-G-FC | - | - | - | - | - | - |
| ADNI-LAN | CC-3-FC | - | - | - | - | - | - |
| ADNI-LAN | CC-4-FC | - | - | - | - | - | - |
| ADNI-LAN | CC-5-FC | - | - | - | - | - | - |
| ADNI-LAN | CC-6-FC | - | - | - | - | - | - |
| ADNI-LAN | CC-7-FC | - | - | - | - | - | - |
| ADNI-LAN | CA-FC | - | - | - | - | - | - |
| ADNI-LAN | CG-FC | - | - | - | - | - | - |
| ADNI-VS | NfL | -0.431 | 0.145 | -0.723 | -0.138 | 0.00495 | 0.165 |
| ADNI-VS | GFAP | -0.392 | 0.147 | -0.69 | -0.095 | 0.0112 | 0.132 |
| ADNI-VS | Ptau_217_ | - | - | - | - | - | - |
| ADNI-VS | amyloid-PET | -0.341 | 0.151 | -0.645 | -0.036 | 0.0293 | 0.093 |
| ADNI-VS | tau-PET | -0.416 | 0.146 | -0.711 | -0.121 | 0.00683 | 0.152 |
| ADNI-VS | AD-Cortical Thickness | 0.385 | 0.148 | 0.086 | 0.684 | 0.0128 | 0.127 |
| ADNI-VS | MSMD | - | - | - | - | - | - |
| ADNI-VS | WMHvol | -0.572 | 0.131 | -0.838 | -0.307 | <0.001 | 0.31 |
| ADNI-VS | AF-FD | - | - | - | - | - | - |
| ADNI-VS | UF-FD | - | - | - | - | - | - |
| ADNI-VS | IFO-FD | - | - | - | - | - | - |
| ADNI-VS | MLF-FD | - | - | - | - | - | - |
| ADNI-VS | ILF-FD | - | - | - | - | - | - |
| ADNI-VS | SLF-I-FD | - | - | - | - | - | - |
| ADNI-VS | SLF-II-FD | - | - | - | - | - | - |
| ADNI-VS | SLF-III-FD | - | - | - | - | - | - |
| ADNI-VS | T-PREF-FD | - | - | - | - | - | - |
| ADNI-VS | T-PREM-FD | - | - | - | - | - | - |
| ADNI-VS | T-PREC-FD | - | - | - | - | - | - |
| ADNI-VS | T-POSTC-FD | - | - | - | - | - | - |
| ADNI-VS | T-PAR-FD | - | - | - | - | - | - |
| ADNI-VS | T-OCC-FD | - | - | - | - | - | - |
| ADNI-VS | ATR-FD | - | - | - | - | - | - |
| ADNI-VS | STR-FD | - | - | - | - | - | - |
| ADNI-VS | OR-FD | - | - | - | - | - | - |
| ADNI-VS | FPT-FD | - | - | - | - | - | - |
| ADNI-VS | CST-FD | - | - | - | - | - | - |
| ADNI-VS | POPT-FD | - | - | - | - | - | - |
| ADNI-VS | CC-1-FD | - | - | - | - | - | - |
| ADNI-VS | CC-G-FD | - | - | - | - | - | - |
| ADNI-VS | CC-3-FD | - | - | - | - | - | - |
| ADNI-VS | CC-4-FD | - | - | - | - | - | - |
| ADNI-VS | CC-5-FD | - | - | - | - | - | - |
| ADNI-VS | CC-6-FD | - | - | - | - | - | - |
| ADNI-VS | CC-7-FD | - | - | - | - | - | - |
| ADNI-VS | CA-FD | - | - | - | - | - | - |
| ADNI-VS | CG-FD | - | - | - | - | - | - |
| ADNI-VS | AF-FC | - | - | - | - | - | - |
| ADNI-VS | UF-FC | - | - | - | - | - | - |
| ADNI-VS | IFO-FC | - | - | - | - | - | - |
| ADNI-VS | MLF-FC | - | - | - | - | - | - |
| ADNI-VS | ILF-FC | - | - | - | - | - | - |
| ADNI-VS | SLF-I-FC | - | - | - | - | - | - |
| ADNI-VS | SLF-II-FC | - | - | - | - | - | - |
| ADNI-VS | SLF-III-FC | - | - | - | - | - | - |
| ADNI-VS | T-PREF-FC | - | - | - | - | - | - |
| ADNI-VS | T-PREM-FC | - | - | - | - | - | - |
| ADNI-VS | T-PREC-FC | - | - | - | - | - | - |
| ADNI-VS | T-POSTC-FC | - | - | - | - | - | - |
| ADNI-VS | T-PAR-FC | - | - | - | - | - | - |
| ADNI-VS | T-OCC-FC | - | - | - | - | - | - |
| ADNI-VS | ATR-FC | - | - | - | - | - | - |
| ADNI-VS | STR-FC | - | - | - | - | - | - |
| ADNI-VS | OR-FC | - | - | - | - | - | - |
| ADNI-VS | FPT-FC | - | - | - | - | - | - |
| ADNI-VS | CST-FC | - | - | - | - | - | - |
| ADNI-VS | POPT-FC | - | - | - | - | - | - |
| ADNI-VS | CC-1-FC | - | - | - | - | - | - |
| ADNI-VS | CC-G-FC | - | - | - | - | - | - |
| ADNI-VS | CC-3-FC | - | - | - | - | - | - |
| ADNI-VS | CC-4-FC | - | - | - | - | - | - |
| ADNI-VS | CC-5-FC | - | - | - | - | - | - |
| ADNI-VS | CC-6-FC | - | - | - | - | - | - |
| ADNI-VS | CC-7-FC | - | - | - | - | - | - |
| ADNI-VS | CA-FC | - | - | - | - | - | - |
| ADNI-VS | CG-FC | - | - | - | - | - | - |
| ADNI-MEM | NfL | -0.422 | 0.145 | -0.715 | -0.128 | 0.00602 | 0.157 |
| ADNI-MEM | GFAP | -0.478 | 0.141 | -0.762 | -0.193 | 0.00158 | 0.209 |
| ADNI-MEM | Ptau_217_ | -0.551 | 0.134 | -0.821 | -0.281 | <0.001 | 0.286 |
| ADNI-MEM | amyloid-PET | -0.599 | 0.128 | -0.859 | -0.34 | <0.001 | 0.343 |
| ADNI-MEM | tau-PET | -0.401 | 0.147 | -0.698 | -0.104 | 0.00935 | 0.139 |
| ADNI-MEM | AD-Cortical Thickness | 0.367 | 0.149 | 0.065 | 0.668 | 0.0184 | 0.112 |
| ADNI-MEM | MSMD | -0.447 | 0.143 | -0.737 | -0.158 | 0.00336 | 0.18 |
| ADNI-MEM | WMHvol | -0.364 | 0.149 | -0.665 | -0.062 | 0.0194 | 0.11 |
| ADNI-MEM | AF-FD | - | - | - | - | - | - |
| ADNI-MEM | UF-FD | - | - | - | - | - | - |
| ADNI-MEM | IFO-FD | - | - | - | - | - | - |
| ADNI-MEM | MLF-FD | - | - | - | - | - | - |
| ADNI-MEM | ILF-FD | - | - | - | - | - | - |
| ADNI-MEM | SLF-I-FD | - | - | - | - | - | - |
| ADNI-MEM | SLF-II-FD | 0.319 | 0.152 | 0.012 | 0.626 | 0.0424 | 0.078 |
| ADNI-MEM | SLF-III-FD | - | - | - | - | - | - |
| ADNI-MEM | T-PREF-FD | - | - | - | - | - | - |
| ADNI-MEM | T-PREM-FD | - | - | - | - | - | - |
| ADNI-MEM | T-PREC-FD | - | - | - | - | - | - |
| ADNI-MEM | T-POSTC-FD | - | - | - | - | - | - |
| ADNI-MEM | T-PAR-FD | - | - | - | - | - | - |
| ADNI-MEM | T-OCC-FD | - | - | - | - | - | - |
| ADNI-MEM | ATR-FD | - | - | - | - | - | - |
| ADNI-MEM | STR-FD | - | - | - | - | - | - |
| ADNI-MEM | OR-FD | - | - | - | - | - | - |
| ADNI-MEM | FPT-FD | - | - | - | - | - | - |
| ADNI-MEM | CST-FD | - | - | - | - | - | - |
| ADNI-MEM | POPT-FD | - | - | - | - | - | - |
| ADNI-MEM | CC-1-FD | - | - | - | - | - | - |
| ADNI-MEM | CC-G-FD | 0.313 | 0.152 | 0.005 | 0.621 | 0.0463 | 0.075 |
| ADNI-MEM | CC-3-FD | - | - | - | - | - | - |
| ADNI-MEM | CC-4-FD | - | - | - | - | - | - |
| ADNI-MEM | CC-5-FD | - | - | - | - | - | - |
| ADNI-MEM | CC-6-FD | - | - | - | - | - | - |
| ADNI-MEM | CC-7-FD | - | - | - | - | - | - |
| ADNI-MEM | CA-FD | - | - | - | - | - | - |
| ADNI-MEM | CG-FD | - | - | - | - | - | - |
| ADNI-MEM | AF-FC | - | - | - | - | - | - |
| ADNI-MEM | UF-FC | - | - | - | - | - | - |
| ADNI-MEM | IFO-FC | - | - | - | - | - | - |
| ADNI-MEM | MLF-FC | - | - | - | - | - | - |
| ADNI-MEM | ILF-FC | - | - | - | - | - | - |
| ADNI-MEM | SLF-I-FC | - | - | - | - | - | - |
| ADNI-MEM | SLF-II-FC | - | - | - | - | - | - |
| ADNI-MEM | SLF-III-FC | - | - | - | - | - | - |
| ADNI-MEM | T-PREF-FC | - | - | - | - | - | - |
| ADNI-MEM | T-PREM-FC | - | - | - | - | - | - |
| ADNI-MEM | T-PREC-FC | - | - | - | - | - | - |
| ADNI-MEM | T-POSTC-FC | - | - | - | - | - | - |
| ADNI-MEM | T-PAR-FC | - | - | - | - | - | - |
| ADNI-MEM | T-OCC-FC | - | - | - | - | - | - |
| ADNI-MEM | ATR-FC | - | - | - | - | - | - |
| ADNI-MEM | STR-FC | - | - | - | - | - | - |
| ADNI-MEM | OR-FC | - | - | - | - | - | - |
| ADNI-MEM | FPT-FC | - | - | - | - | - | - |
| ADNI-MEM | CST-FC | - | - | - | - | - | - |
| ADNI-MEM | POPT-FC | - | - | - | - | - | - |
| ADNI-MEM | CC-1-FC | - | - | - | - | - | - |
| ADNI-MEM | CC-G-FC | - | - | - | - | - | - |
| ADNI-MEM | CC-3-FC | - | - | - | - | - | - |
| ADNI-MEM | CC-4-FC | - | - | - | - | - | - |
| ADNI-MEM | CC-5-FC | - | - | - | - | - | - |
| ADNI-MEM | CC-6-FC | - | - | - | - | - | - |
| ADNI-MEM | CC-7-FC | - | - | - | - | - | - |
| ADNI-MEM | CA-FC | - | - | - | - | - | - |
| ADNI-MEM | CG-FC | - | - | - | - | - | - |
| ADNI-EF2 | NfL | -0.489 | 0.14 | -0.772 | -0.207 | 0.00117 | 0.22 |
| ADNI-EF2 | GFAP | -0.573 | 0.131 | -0.838 | -0.308 | <0.001 | 0.311 |
| ADNI-EF2 | Ptau_217_ | -0.493 | 0.139 | -0.774 | -0.211 | 0.00107 | 0.223 |
| ADNI-EF2 | amyloid-PET | -0.574 | 0.131 | -0.839 | -0.308 | <0.001 | 0.312 |
| ADNI-EF2 | tau-PET | -0.412 | 0.146 | -0.707 | -0.117 | 0.00747 | 0.148 |
| ADNI-EF2 | AD-Cortical Thickness | 0.535 | 0.135 | 0.262 | 0.809 | <0.001 | 0.268 |
| ADNI-EF2 | MSMD | -0.36 | 0.149 | -0.662 | -0.057 | 0.021 | 0.107 |
| ADNI-EF2 | WMHvol | -0.531 | 0.136 | -0.806 | -0.257 | <0.001 | 0.264 |
| ADNI-EF2 | AF-FD | 0.412 | 0.146 | 0.117 | 0.707 | 0.00742 | 0.149 |
| ADNI-EF2 | UF-FD | - | - | - | - | - | - |
| ADNI-EF2 | IFO-FD | 0.484 | 0.14 | 0.201 | 0.767 | 0.00135 | 0.215 |
| ADNI-EF2 | MLF-FD | 0.402 | 0.147 | 0.106 | 0.699 | 0.00913 | 0.14 |
| ADNI-EF2 | ILF-FD | 0.513 | 0.137 | 0.235 | 0.791 | <0.001 | 0.244 |
| ADNI-EF2 | SLF-I-FD | - | - | - | - | - | - |
| ADNI-EF2 | SLF-II-FD | 0.392 | 0.147 | 0.094 | 0.69 | 0.0112 | 0.132 |
| ADNI-EF2 | SLF-III-FD | 0.324 | 0.151 | 0.018 | 0.631 | 0.0386 | 0.082 |
| ADNI-EF2 | T-PREF-FD | 0.418 | 0.145 | 0.124 | 0.712 | 0.0065 | 0.154 |
| ADNI-EF2 | T-PREM-FD | 0.449 | 0.143 | 0.16 | 0.738 | 0.00323 | 0.181 |
| ADNI-EF2 | T-PREC-FD | - | - | - | - | - | - |
| ADNI-EF2 | T-POSTC-FD | - | - | - | - | - | - |
| ADNI-EF2 | T-PAR-FD | 0.354 | 0.15 | 0.051 | 0.657 | 0.0232 | 0.103 |
| ADNI-EF2 | T-OCC-FD | 0.456 | 0.143 | 0.168 | 0.744 | 0.00274 | 0.188 |
| ADNI-EF2 | ATR-FD | 0.517 | 0.137 | 0.24 | 0.794 | <0.001 | 0.249 |
| ADNI-EF2 | STR-FD | - | - | - | - | - | - |
| ADNI-EF2 | OR-FD | 0.44 | 0.144 | 0.149 | 0.731 | 0.00397 | 0.173 |
| ADNI-EF2 | FPT-FD | 0.328 | 0.151 | 0.022 | 0.634 | 0.0363 | 0.085 |
| ADNI-EF2 | CST-FD | - | - | - | - | - | - |
| ADNI-EF2 | POPT-FD | - | - | - | - | - | - |
| ADNI-EF2 | CC-1-FD | 0.536 | 0.135 | 0.262 | 0.809 | <0.001 | 0.269 |
| ADNI-EF2 | CC-G-FD | 0.554 | 0.133 | 0.284 | 0.824 | <0.001 | 0.289 |
| ADNI-EF2 | CC-3-FD | 0.571 | 0.131 | 0.305 | 0.837 | <0.001 | 0.309 |
| ADNI-EF2 | CC-4-FD | 0.463 | 0.142 | 0.176 | 0.75 | 0.00231 | 0.194 |
| ADNI-EF2 | CC-5-FD | 0.43 | 0.145 | 0.137 | 0.722 | 0.00507 | 0.164 |
| ADNI-EF2 | CC-6-FD | 0.451 | 0.143 | 0.162 | 0.74 | 0.00308 | 0.183 |
| ADNI-EF2 | CC-7-FD | 0.457 | 0.142 | 0.169 | 0.745 | 0.00264 | 0.189 |
| ADNI-EF2 | CA-FD | 0.413 | 0.146 | 0.117 | 0.708 | 0.00736 | 0.149 |
| ADNI-EF2 | CG-FD | 0.324 | 0.151 | 0.018 | 0.631 | 0.0387 | 0.082 |
| ADNI-EF2 | AF-FC | - | - | - | - | - | - |
| ADNI-EF2 | UF-FC | - | - | - | - | - | - |
| ADNI-EF2 | IFO-FC | - | - | - | - | - | - |
| ADNI-EF2 | MLF-FC | - | - | - | - | - | - |
| ADNI-EF2 | ILF-FC | - | - | - | - | - | - |
| ADNI-EF2 | SLF-I-FC | - | - | - | - | - | - |
| ADNI-EF2 | SLF-II-FC | - | - | - | - | - | - |
| ADNI-EF2 | SLF-III-FC | - | - | - | - | - | - |
| ADNI-EF2 | T-PREF-FC | - | - | - | - | - | - |
| ADNI-EF2 | T-PREM-FC | - | - | - | - | - | - |
| ADNI-EF2 | T-PREC-FC | - | - | - | - | - | - |
| ADNI-EF2 | T-POSTC-FC | - | - | - | - | - | - |
| ADNI-EF2 | T-PAR-FC | - | - | - | - | - | - |
| ADNI-EF2 | T-OCC-FC | - | - | - | - | - | - |
| ADNI-EF2 | ATR-FC | - | - | - | - | - | - |
| ADNI-EF2 | STR-FC | - | - | - | - | - | - |
| ADNI-EF2 | OR-FC | - | - | - | - | - | - |
| ADNI-EF2 | FPT-FC | - | - | - | - | - | - |
| ADNI-EF2 | CST-FC | - | - | - | - | - | - |
| ADNI-EF2 | POPT-FC | - | - | - | - | - | - |
| ADNI-EF2 | CC-1-FC | - | - | - | - | - | - |
| ADNI-EF2 | CC-G-FC | - | - | - | - | - | - |
| ADNI-EF2 | CC-3-FC | - | - | - | - | - | - |
| ADNI-EF2 | CC-4-FC | - | - | - | - | - | - |
| ADNI-EF2 | CC-5-FC | - | - | - | - | - | - |
| ADNI-EF2 | CC-6-FC | - | - | - | - | - | - |
| ADNI-EF2 | CC-7-FC | - | - | - | - | - | - |
| ADNI-EF2 | CA-FC | 0.402 | 0.147 | 0.105 | 0.698 | 0.00927 | 0.14 |
| ADNI-EF2 | CG-FC | - | - | - | - | - | - |
| ADAS-13 | NfL | 0.461 | 0.142 | 0.174 | 0.749 | 0.0024 | 0.193 |
| ADAS-13 | GFAP | 0.521 | 0.137 | 0.245 | 0.798 | <0.001 | 0.253 |
| ADAS-13 | Ptau_217_ | 0.587 | 0.13 | 0.324 | 0.849 | <0.001 | 0.327 |
| ADAS-13 | amyloid-PET | 0.592 | 0.129 | 0.332 | 0.853 | <0.001 | 0.334 |
| ADAS-13 | tau-PET | 0.516 | 0.137 | 0.238 | 0.793 | <0.001 | 0.247 |
| ADAS-13 | AD-Cortical Thickness | -0.51 | 0.138 | -0.789 | -0.231 | <0.001 | 0.241 |
| ADAS-13 | MSMD | 0.395 | 0.147 | 0.097 | 0.692 | 0.0106 | 0.134 |
| ADAS-13 | WMHvol | 0.44 | 0.144 | 0.149 | 0.731 | 0.00401 | 0.173 |
| ADAS-13 | AF-FD | - | - | - | - | - | - |
| ADAS-13 | UF-FD | - | - | - | - | - | - |
| ADAS-13 | IFO-FD | - | - | - | - | - | - |
| ADAS-13 | MLF-FD | - | - | - | - | - | - |
| ADAS-13 | ILF-FD | - | - | - | - | - | - |
| ADAS-13 | SLF-I-FD | - | - | - | - | - | - |
| ADAS-13 | SLF-II-FD | - | - | - | - | - | - |
| ADAS-13 | SLF-III-FD | - | - | - | - | - | - |
| ADAS-13 | T-PREF-FD | - | - | - | - | - | - |
| ADAS-13 | T-PREM-FD | - | - | - | - | - | - |
| ADAS-13 | T-PREC-FD | - | - | - | - | - | - |
| ADAS-13 | T-POSTC-FD | - | - | - | - | - | - |
| ADAS-13 | T-PAR-FD | - | - | - | - | - | - |
| ADAS-13 | T-OCC-FD | - | - | - | - | - | - |
| ADAS-13 | ATR-FD | - | - | - | - | - | - |
| ADAS-13 | STR-FD | - | - | - | - | - | - |
| ADAS-13 | OR-FD | - | - | - | - | - | - |
| ADAS-13 | FPT-FD | - | - | - | - | - | - |
| ADAS-13 | CST-FD | - | - | - | - | - | - |
| ADAS-13 | POPT-FD | - | - | - | - | - | - |
| ADAS-13 | CC-1-FD | -0.316 | 0.152 | -0.623 | -0.008 | 0.0444 | 0.077 |
| ADAS-13 | CC-G-FD | - | - | - | - | - | - |
| ADAS-13 | CC-3-FD | - | - | - | - | - | - |
| ADAS-13 | CC-4-FD | - | - | - | - | - | - |
| ADAS-13 | CC-5-FD | - | - | - | - | - | - |
| ADAS-13 | CC-6-FD | - | - | - | - | - | - |
| ADAS-13 | CC-7-FD | - | - | - | - | - | - |
| ADAS-13 | CA-FD | - | - | - | - | - | - |
| ADAS-13 | CG-FD | - | - | - | - | - | - |
| ADAS-13 | AF-FC | - | - | - | - | - | - |
| ADAS-13 | UF-FC | - | - | - | - | - | - |
| ADAS-13 | IFO-FC | - | - | - | - | - | - |
| ADAS-13 | MLF-FC | - | - | - | - | - | - |
| ADAS-13 | ILF-FC | - | - | - | - | - | - |
| ADAS-13 | SLF-I-FC | - | - | - | - | - | - |
| ADAS-13 | SLF-II-FC | - | - | - | - | - | - |
| ADAS-13 | SLF-III-FC | - | - | - | - | - | - |
| ADAS-13 | T-PREF-FC | - | - | - | - | - | - |
| ADAS-13 | T-PREM-FC | - | - | - | - | - | - |
| ADAS-13 | T-PREC-FC | - | - | - | - | - | - |
| ADAS-13 | T-POSTC-FC | - | - | - | - | - | - |
| ADAS-13 | T-PAR-FC | - | - | - | - | - | - |
| ADAS-13 | T-OCC-FC | - | - | - | - | - | - |
| ADAS-13 | ATR-FC | - | - | - | - | - | - |
| ADAS-13 | STR-FC | - | - | - | - | - | - |
| ADAS-13 | OR-FC | - | - | - | - | - | - |
| ADAS-13 | FPT-FC | - | - | - | - | - | - |
| ADAS-13 | CST-FC | - | - | - | - | - | - |
| ADAS-13 | POPT-FC | - | - | - | - | - | - |
| ADAS-13 | CC-1-FC | - | - | - | - | - | - |
| ADAS-13 | CC-G-FC | - | - | - | - | - | - |
| ADAS-13 | CC-3-FC | - | - | - | - | - | - |
| ADAS-13 | CC-4-FC | - | - | - | - | - | - |
| ADAS-13 | CC-5-FC | - | - | - | - | - | - |
| ADAS-13 | CC-6-FC | - | - | - | - | - | - |
| ADAS-13 | CC-7-FC | - | - | - | - | - | - |
| ADAS-13 | CA-FC | - | - | - | - | - | - |
| ADAS-13 | CG-FC | - | - | - | - | - | - |
| MMSE | NfL | - | - | - | - | - | - |
| MMSE | GFAP | - | - | - | - | - | - |
| MMSE | Ptau_217_ | -0.336 | 0.151 | -0.641 | -0.031 | 0.0315 | 0.09 |
| MMSE | amyloid-PET | -0.598 | 0.128 | -0.858 | -0.339 | <0.001 | 0.341 |
| MMSE | tau-PET | -0.352 | 0.15 | -0.655 | -0.049 | 0.0239 | 0.102 |
| MMSE | AD-Cortical Thickness | 0.364 | 0.149 | 0.063 | 0.666 | 0.0192 | 0.111 |
| MMSE | MSMD | - | - | - | - | - | - |
| MMSE | WMHvol | - | - | - | - | - | - |
| MMSE | AF-FD | - | - | - | - | - | - |
| MMSE | UF-FD | - | - | - | - | - | - |
| MMSE | IFO-FD | - | - | - | - | - | - |
| MMSE | MLF-FD | - | - | - | - | - | - |
| MMSE | ILF-FD | - | - | - | - | - | - |
| MMSE | SLF-I-FD | - | - | - | - | - | - |
| MMSE | SLF-II-FD | - | - | - | - | - | - |
| MMSE | SLF-III-FD | - | - | - | - | - | - |
| MMSE | T-PREF-FD | - | - | - | - | - | - |
| MMSE | T-PREM-FD | - | - | - | - | - | - |
| MMSE | T-PREC-FD | - | - | - | - | - | - |
| MMSE | T-POSTC-FD | - | - | - | - | - | - |
| MMSE | T-PAR-FD | - | - | - | - | - | - |
| MMSE | T-OCC-FD | - | - | - | - | - | - |
| MMSE | ATR-FD | - | - | - | - | - | - |
| MMSE | STR-FD | - | - | - | - | - | - |
| MMSE | OR-FD | - | - | - | - | - | - |
| MMSE | FPT-FD | - | - | - | - | - | - |
| MMSE | CST-FD | - | - | - | - | - | - |
| MMSE | POPT-FD | - | - | - | - | - | - |
| MMSE | CC-1-FD | - | - | - | - | - | - |
| MMSE | CC-G-FD | - | - | - | - | - | - |
| MMSE | CC-3-FD | - | - | - | - | - | - |
| MMSE | CC-4-FD | - | - | - | - | - | - |
| MMSE | CC-5-FD | - | - | - | - | - | - |
| MMSE | CC-6-FD | - | - | - | - | - | - |
| MMSE | CC-7-FD | - | - | - | - | - | - |
| MMSE | CA-FD | - | - | - | - | - | - |
| MMSE | CG-FD | - | - | - | - | - | - |
| MMSE | AF-FC | - | - | - | - | - | - |
| MMSE | UF-FC | - | - | - | - | - | - |
| MMSE | IFO-FC | - | - | - | - | - | - |
| MMSE | MLF-FC | - | - | - | - | - | - |
| MMSE | ILF-FC | - | - | - | - | - | - |
| MMSE | SLF-I-FC | - | - | - | - | - | - |
| MMSE | SLF-II-FC | - | - | - | - | - | - |
| MMSE | SLF-III-FC | - | - | - | - | - | - |
| MMSE | T-PREF-FC | - | - | - | - | - | - |
| MMSE | T-PREM-FC | - | - | - | - | - | - |
| MMSE | T-PREC-FC | - | - | - | - | - | - |
| MMSE | T-POSTC-FC | - | - | - | - | - | - |
| MMSE | T-PAR-FC | - | - | - | - | - | - |
| MMSE | T-OCC-FC | - | - | - | - | - | - |
| MMSE | ATR-FC | - | - | - | - | - | - |
| MMSE | STR-FC | - | - | - | - | - | - |
| MMSE | OR-FC | - | - | - | - | - | - |
| MMSE | FPT-FC | - | - | - | - | - | - |
| MMSE | CST-FC | - | - | - | - | - | - |
| MMSE | POPT-FC | - | - | - | - | - | - |
| MMSE | CC-1-FC | - | - | - | - | - | - |
| MMSE | CC-G-FC | - | - | - | - | - | - |
| MMSE | CC-3-FC | - | - | - | - | - | - |
| MMSE | CC-4-FC | - | - | - | - | - | - |
| MMSE | CC-5-FC | - | - | - | - | - | - |
| MMSE | CC-6-FC | - | - | - | - | - | - |
| MMSE | CC-7-FC | - | - | - | - | - | - |
| MMSE | CA-FC | - | - | - | - | - | - |
| MMSE | CG-FC | - | - | - | - | - | - |
| MoCA | NfL | -0.447 | 0.143 | -0.737 | -0.158 | 0.00338 | 0.184 |
| MoCA | GFAP | -0.546 | 0.134 | -0.817 | -0.274 | <0.001 | 0.285 |
| MoCA | Ptau_217_ | -0.54 | 0.135 | -0.812 | -0.268 | <0.001 | 0.279 |
| MoCA | amyloid-PET | -0.575 | 0.132 | -0.843 | -0.308 | <0.001 | 0.315 |
| MoCA | tau-PET | -0.389 | 0.147 | -0.687 | -0.09 | 0.012 | 0.133 |
| MoCA | AD-Cortical Thickness | 0.343 | 0.151 | 0.037 | 0.649 | 0.0289 | 0.096 |
| MoCA | MSMD | - | - | - | - | - | - |
| MoCA | WMHvol | -0.38 | 0.148 | -0.68 | -0.081 | 0.0142 | 0.126 |
| MoCA | AF-FD | - | - | - | - | - | - |
| MoCA | UF-FD | - | - | - | - | - | - |
| MoCA | IFO-FD | - | - | - | - | - | - |
| MoCA | MLF-FD | - | - | - | - | - | - |
| MoCA | ILF-FD | - | - | - | - | - | - |
| MoCA | SLF-I-FD | - | - | - | - | - | - |
| MoCA | SLF-II-FD | - | - | - | - | - | - |
| MoCA | SLF-III-FD | - | - | - | - | - | - |
| MoCA | T-PREF-FD | - | - | - | - | - | - |
| MoCA | T-PREM-FD | - | - | - | - | - | - |
| MoCA | T-PREC-FD | - | - | - | - | - | - |
| MoCA | T-POSTC-FD | - | - | - | - | - | - |
| MoCA | T-PAR-FD | - | - | - | - | - | - |
| MoCA | T-OCC-FD | - | - | - | - | - | - |
| MoCA | ATR-FD | - | - | - | - | - | - |
| MoCA | STR-FD | - | - | - | - | - | - |
| MoCA | OR-FD | - | - | - | - | - | - |
| MoCA | FPT-FD | - | - | - | - | - | - |
| MoCA | CST-FD | - | - | - | - | - | - |
| MoCA | POPT-FD | - | - | - | - | - | - |
| MoCA | CC-1-FD | - | - | - | - | - | - |
| MoCA | CC-G-FD | - | - | - | - | - | - |
| MoCA | CC-3-FD | - | - | - | - | - | - |
| MoCA | CC-4-FD | - | - | - | - | - | - |
| MoCA | CC-5-FD | - | - | - | - | - | - |
| MoCA | CC-6-FD | - | - | - | - | - | - |
| MoCA | CC-7-FD | - | - | - | - | - | - |
| MoCA | CA-FD | - | - | - | - | - | - |
| MoCA | CG-FD | - | - | - | - | - | - |
| MoCA | AF-FC | - | - | - | - | - | - |
| MoCA | UF-FC | - | - | - | - | - | - |
| MoCA | IFO-FC | - | - | - | - | - | - |
| MoCA | MLF-FC | - | - | - | - | - | - |
| MoCA | ILF-FC | - | - | - | - | - | - |
| MoCA | SLF-I-FC | - | - | - | - | - | - |
| MoCA | SLF-II-FC | - | - | - | - | - | - |
| MoCA | SLF-III-FC | - | - | - | - | - | - |
| MoCA | T-PREF-FC | - | - | - | - | - | - |
| MoCA | T-PREM-FC | - | - | - | - | - | - |
| MoCA | T-PREC-FC | - | - | - | - | - | - |
| MoCA | T-POSTC-FC | - | - | - | - | - | - |
| MoCA | T-PAR-FC | - | - | - | - | - | - |
| MoCA | T-OCC-FC | - | - | - | - | - | - |
| MoCA | ATR-FC | - | - | - | - | - | - |
| MoCA | STR-FC | - | - | - | - | - | - |
| MoCA | OR-FC | - | - | - | - | - | - |
| MoCA | FPT-FC | - | - | - | - | - | - |
| MoCA | CST-FC | - | - | - | - | - | - |
| MoCA | POPT-FC | - | - | - | - | - | - |
| MoCA | CC-1-FC | - | - | - | - | - | - |
| MoCA | CC-G-FC | - | - | - | - | - | - |
| MoCA | CC-3-FC | - | - | - | - | - | - |
| MoCA | CC-4-FC | - | - | - | - | - | - |
| MoCA | CC-5-FC | - | - | - | - | - | - |
| MoCA | CC-6-FC | - | - | - | - | - | - |
| MoCA | CC-7-FC | - | - | - | - | - | - |
| MoCA | CA-FC | - | - | - | - | - | - |
| MoCA | CG-FC | - | - | - | - | - | - |
